# Maternal Age at Menarche Genes Determines Fetal Growth Restriction Risk

**DOI:** 10.3390/ijms25052647

**Published:** 2024-02-24

**Authors:** Evgeny Reshetnikov, Maria Churnosova, Yuliya Reshetnikova, Vadim Stepanov, Anna Bocharova, Victoria Serebrova, Ekaterina Trifonova, Irina Ponomarenko, Inna Sorokina, Olga Efremova, Valentina Orlova, Irina Batlutskaya, Marina Ponomarenko, Vladimir Churnosov, Inna Aristova, Alexey Polonikov, Mikhail Churnosov

**Affiliations:** 1 Department of Medical Biological Disciplines, Belgorod State National Research University, 308015 Belgorod, Russia; reshetnikov@bsu.edu.ru (E.R.); churnosovamary@gmail.com (M.C.); 130226@bsu.edu.ru (Y.R.); ponomarenko_i@bsu.edu.ru (I.P.); sorokina@bsu.edu.ru (I.S.); efremova@bsu.edu.ru (O.E.); orlova@bsu.edu.ru (V.O.); bat@bsu.edu.ru (I.B.); 1256888@bsu.edu.ru (M.P.); churnosov_v@bsu.edu.ru (V.C.); aristova@bsu.edu.ru (I.A.); polonikov@rambler.ru (A.P.); 2 Research Institute for Medical Genetics, Tomsk National Research Medical Center of the Russian Academy of Sciences, 634050 Tomsk, Russia; vadim.stepanov@medgenetics.ru (V.S.); anna.bocharova@medgenetics.ru (A.B.); vika.serebrova@medgenetics.ru (V.S.); ekaterina.trifonova@medgenetics.ru (E.T.); 3 Department of Biology, Medical Genetics and Ecology and Research Institute for Genetic and Molecular Epidemiology, Kursk State Medical University, 305041 Kursk, Russia

**Keywords:** fetal growth restriction, single nucleotide polymorphism, association, age at menarche

## Abstract

We aimed to explore the potential link of maternal age at menarche (mAAM) gene polymorphisms with risk of the fetal growth restriction (FGR). This case (FGR)–control (FGR free) study included 904 women (273 FGR and 631 control) in the third trimester of gestation examined/treated in the Departments of Obstetrics. For single nucleotide polymorphism (SNP) multiplex genotyping, 50 candidate loci of mAAM were chosen. The relationship of mAAM SNPs and FGR was appreciated by regression procedures (logistic/model-based multifactor dimensionality reduction [MB-MDR]) with subsequent in silico assessment of the assumed functionality pithy of FGR-related loci. Three mAAM-appertain loci were FGR-linked to genes such as *KISS1* (rs7538038) (effect allele G-odds ratio (OR)_allelic_ = 0.63/p_perm_ = 0.0003; OR_additive_ = 0.61/p_perm_ = 0.001; OR_dominant_ = 0.56/p_perm_ = 0.001), *NKX2-1* (rs999460) (effect allele A-OR_allelic_ = 1.37/p_perm_ = 0.003; OR_additive_ = 1.45/p_perm_ = 0.002; OR_recessive_ = 2.41/p_perm_ = 0.0002), *GPRC5B* (rs12444979) (effect allele T-OR_allelic_ = 1.67/p_perm_ = 0.0003; OR_dominant_ = 1.59/p_perm_ = 0.011; OR_additive_ = 1.56/p_perm_ = 0.009). The haplotype ACA *FSHB* gene (rs555621*rs11031010*rs1782507) was FRG-correlated (OR = 0.71/p_perm_ = 0.05). Ten FGR-implicated interworking models were founded for 13 SNPs (p_perm_ ≤ 0.001). The rs999460 *NKX2-1* and rs12444979 *GPRC5B* interplays significantly influenced the FGR risk (these SNPs were present in 50% of models). FGR-related mAAM-appertain 15 polymorphic variants and 350 linked SNPs were functionally momentous in relation to 39 genes participating in the regulation of hormone levels, the ovulation cycle process, male gonad development and vitamin D metabolism. Thus, this study showed, for the first time, that the mAAM-appertain genes determine FGR risk.

## 1. Introduction

Fetal growth restriction (FGR) is defined as a pathological inhibition of fetal intrauterine growth and the inability of the fetus to reach its growth potential, in which fetal size is below the 10th percentile for a given gestational age [1,2]. FGR is a common complication of pregnancy, the incidence of which reaches up to 10% worldwide [1,3]. FGR is the leading cause of stillbirth (more than 50% of stillborn infants had FGR or were small for gestational age) and neonatal mortality/morbidity [4,5,6,7], and the long-term effects of FGR in adulthood are associated with an increased risk of developing cardiovascular/metabolic disorders (dyslipidemia; insulin resistance; arterial hypertension; obesity; type II diabetes; fatty liver, etc.) [8,9,10].

The FGR pathogenesis is complex and can be associated with various factors (maternal; placental; fetal), leading to a limited supply of nutrients and oxygen to the fetus [3,11,12,13,14,15,16,17]. Maternal risk factors such as advanced age, underweight, hypertension, diabetes, genetics, etc., are essential in the formation of FGR [2,18]. The above violations of the maternal organism can facilitate the evolution of various placental disorders (vascular malperfusion disturbances in the maternal–fetal system (fibrin deposition/infarction/chronic abruption), chronic inflammation of placenta, etc.), leading to change in placental nutrient transport (glucose; amino acids; fatty acids; oxygen intake) which in turn can cause the formation of FGR [2,18,19].

Among the indicators reflecting the state of a woman’s reproductive system (hypothalamic–pituitary–ovarian system/axis functioning) and her possible health problems in the future life is the age at menarche (AAM) [20,21]. AAM correlates significantly with hormone-, glucose/insulin-dependent pathways in a woman’s body [20,21], and, as a result, can affect the course/complications of pregnancy (e.g., blood pressure, preeclampsia, premature birth, gestational diabetes) [22,23,24,25,26,27]. Also, such AAM-mediated signs/diseases as body mass index (BMI)/obesity/cardiovascular diseases, etc. [20,28], can have a weighty aftermath for the course/outcomes of pregnancy [18]. Previous studies have shown the association of maternal mAAM (mAAM) with premature birth [24], pregnant blood pressure [25] and preeclampsia [22,23,26], as well as offspring BMI [29,30]. Importantly, all the abovementioned pregnancy course/outcomes directly correlate with FGR [2,18]. AAM gene association with female reproductive organ diseases [31,32,33], BMI-related phenotypes (height; weight; BMI) of adults [34,35,36,37,38,39], newborn weight [40], and complications of pregnancy (gestational diabetes) [27] has been demonstrated. Notwithstanding, the relationship of AAM genes with FGR has not been studied to date. We aimed to explore the potential link of mAAM gene polymorphisms with FGR risk.

## 2. Results

### 2.1. FGR and FGR Free Group Characteristics

All basic medical/anamnestic/biological characteristics of FGR and FGR free groups from matched/unmatched data are shown in Table 1. According to the information presented in Table 1, such parameters as age (*p* = 0.017), pre-pregnancy maternal BMI (mBMI) parameter distribution (*p* = 0.0001), number of gravidity (*p* = 0.004) and induced abortions (*p* = 0.0001) in the anamnesis, the presence in anamnesis of arterial hypertension (*p* = 0.0001), FGR (*p* = 0.00001) and preeclampsia (*p* = 0.001) between the two cohorts compared were statistically different, and therefore these characteristics were included in the logistic regression models as confounders.

### 2.2. SNP/Haplotype Association Analysis

All 49 single nucleotide polymorphisms (SNPs) were consistent with Hardy–Weinberg Equilibrium (HWE) (all *p*-values were ≥0.022 at a threshold value for 49 SNPs p_bonf_ = 0.05/49 = 0.001) (Table S1). Three mAAM-appertain loci were FGR-linked, including such genes as *KISS1* (rs7538038) (effect allele G-odds ratio (OR)_allelic_ = 0.63/*p* = 0.0003/p_perm_ = 0.0003; OR_additive_ = 0.61/*p* = 0.001/p_perm_ = 0.001/power = 96.00%; OR_dominant_ = 0.56/*p* = 0.001/p_perm_ = 0.001/power = 96.27%, *NKX2-1* (rs999460) (effect allele A-OR_allelic_ = 1.37/*p* = 0.003/p_perm_ = 0.003; OR_additive_ = 1.45/*p* = 0.002/p_perm_ = 0.002/power = 93.92%; OR_recessive_ = 2.41/*p* = 0.0002/p_perm_ = 0.0002/power = 99.44%), *GPRC5B* (rs12444979) (effect allele T-OR_allelic_ = 1.67/*p* = 0.0002/p_perm_ = 0.0003; OR_dominant_ = 1.59/*p* = 0.011/p_perm_ = 0.011/power = 83.75%; OR_additive_ = 1.56/*p* = 0.005/p_perm_ = 0.009/power = 89.88%) (Table 2). The haplotype ACA *FSHB* gene (rs555621*rs11031010*rs1782507) was FRG-correlated (OR = 0.71/*p* = 0.017/p_perm_ = 0.05) (Table 3).

**Table 1:** Phenotypic characteristics of study participants.

Parameters	FGR Patients $\bar{X}$ ± SD/%(n)	Controls $\bar{X}$ ± SD/%(n)	*p*-Value
N	273	631	
Age, years	27.18 ± 4.84	26.57 ± 4.94	**0.017**
Height, m	1.65 ± 0.06	1.65 ± 0.06	0.52
Weight, kg	63.53 ± 11.81	63.50 ± 11.22	0.78
Pre-pregnancy BMI, kg/m^2^	23.40 ± 4.31	23.40 ± 3.47	0.23
Underweight (<18.50) Normal weight (18.50–24.99) Overweight (25.00–29.99) Obesity (>30)	8.69 (24) 66.30 (181) 14.29 (39) 10.62 (29)	5.55 (35) 66.72 (421) 22.19 (140) 5.55 (35)	**0.0001**
Age at menarche and menstrual cycle			
Age at menarche, years	12.80 ± 1.14	12.83 ± 1.17	0.51
Early (<12) Average (12–14) Late (>14)	3.66 (10) 90.11 (246) 6.23 (17)	6.5 (41) 86.69 (547) 6.81 (43)	0.23
Menstrual cycle length, days	28.11 ± 1.64	28.05 ± 1.93	0.19
Duration of menstrual bleeding, days	5.02 ± 0.85	5.04 ± 1.03	0.93
Reproductive characteristic			
First pregnancy	34.93 (95)	40.41 (255)	0.19
No. of gravidity	1.47 ± 1.55	1.07 ± 2.04	**0.004**
No. of births	0.59 ± 0.69	0.56 ± 0.81	0.43
Stillbirth	0.01 ± 0.10	0.007 ± 0.08	0.62
No. of spontaneous abortions	0.23 ± 0.53	0.13 ± 0.35	0.07
No. of induced abortions	0.64 ± 0.99	0.37 ± 0.78	**0.0001**
Risk factors			
Smoking *	61.54 (168)	63.71 (402)	0.87
Alcohol **	81.68 (223)	79.72 (503)	0.79
History of arterial hypertension	8.79 (24)	1.74 (11)	**0.0001**
History of sexually transmitted diseases	35.56 (16)	26.62 (168)	0.28
History of preeclampsia	10.99 (30)	3.80 (24)	**0.001**
History of FGR	19.78 (54)	0.79 (5)	**0.00001**

Note: BMI, Body mass index; significant *p*-values showed in bold; *—regular and irregular (episodic) smoking at least 1 time or more per week; **—drinking low-alcohol drinks (wine, beer and others) or/and strong alcoholic beverages at least 1 time or more per week; *p* values < 0.05 are shown in bold.

### 2.3. Association Analysis of SNP Interactions

Ten FGR-risky/protective interworking models were founded for 13 SNPs (out of 49 examined loci) (p_perm_ ≤ 0.001) (Table 4). The rs999460 *NKX2-1* and rs12444979 *GPRC5B* interplays significantly influenced FGR risk (these SNPs were present in 50% models [five models each] and their two-SNP interplay was part of three models [30% models] of a three-order interaction). The maximum effect on FGR risk was registered by us for a model that includes such four mAAM polymorphisms as rs1544410 *VDR-*rs1398217 *SKOR2-*rs7579411 *LHCGR-*rs314280 *LIN28B* (WH = 49.59/*p* = 1.89 × 10^−12^/p_perm_ < 0.001) (Table 4). The highest degree of statistical magnitude of associations was registered for such genetic combinations as rs222003 GG *GC*×rs7538038 AG *KISS1*×rs999460 GA *NKX2-1* (*beta(FGR)* = −1.07; *p* = 0.00004), rs3020394 AA *ESR1*×rs12444979 CC *GPRC5B* (*beta(FGR)* = −0.57; *p* = 0.0003), rs12444979 CC *GPRC5B*×rs999460 GG *NKX2-1*×rs11031010 CC *FSHB* (*beta(FGR)* = −0.64; *p* = 0.0003) (protective effects) and rs7538038 AA *KISS1*×rs999460 AA *NKX2-1* (*beta(FGR)* = 0.98; *p* = 0.0004), rs1544410 GA *VDR*×rs1398217 CG *SKOR2*×rs7579411 CC *LHCGR*×rs314280 CT *LIN28B* (*beta(FGR)* = 1.26; *p* = 0.0004) (risk effects) (Table S2).

**Table 2:** Associations of 49 mAAM-involved SNPs with FGR.

Minore Allele (SNP)	Gene	Chr	N	Genetic Models															
				Allelic				Additive				Dominant				Recessive			
				OR	95%CI		*p*	OR	95%CI		*p*	OR	95%CI		*p*	OR	95%CI		*p*
					L95	U95			L95	U95			L95	U95			L95	U95	
T (rs1514175)	*TNNI3K*	1	896	1.08	0.88	1.33	0.454	1.04	0.83	1.30	0.759	1.04	0.75	1.44	0.827	1.07	0.69	1.65	0.764
T (rs466639)	*RXRG*	1	899	0.91	0.66	1.24	0.534	0.89	0.63	1.25	0.505	0.90	0.61	1.33	0.613	0.65	0.20	2.08	0.464
G (rs7538038)	*KISS1*	1	**898**	**0.63**	**0.49**	**0.81**	**0.0002**	**0.61**	**0.46**	**0.82**	**0.001**	**0.56**	**0.40**	**0.79**	**0.001**	0.57	0.24	1.37	0.208
C (rs713586)	*RBJ*	2	899	0.97	0.79	1.19	0.775	0.97	0.78	1.21	0.777	0.97	0.69	1.36	0.838	0.95	0.64	1.40	0.790
A (rs2164808)	*POMC*	2	898	1.09	0.89	1.34	0.382	1.06	0.84	1.32	0.641	1.14	0.80	1.62	0.475	1.00	0.68	1.48	0.995
A (rs7589318)	*POMC*	2	900	0.98	0.79	1.22	0.867	0.96	0.75	1.22	0.739	1.16	0.84	1.59	0.364	0.49	0.27	0.91	0.025
C (rs4374421)	*LHCGR*	2	874	0.93	0.74	1.16	0.504	0.94	0.73	1.21	0.637	0.99	0.71	1.37	0.941	0.76	0.42	1.36	0.356
T (rs7579411)	*LHCGR*	2	891	0.91	0.74	1.11	0.351	0.98	0.78	1.25	0.890	0.85	0.60	1.20	0.347	1.19	0.80	1.79	0.392
C (rs4953616)	*LHCGR*	2	892	0.96	0.76	1.20	0.697	0.93	0.72	1.20	0.569	0.89	0.65	1.23	0.478	1.00	0.54	1.84	0.993
G (rs6732220)	*FSHR*	2	898	0.93	0.74	1.17	0.538	0.87	0.67	1.14	0.321	0.93	0.68	1.28	0.664	0.52	0.23	1.14	0.104
G (rs4953655)	*FSHR*	2	898	0.87	0.68	1.10	0.248	0.81	0.61	1.06	0.129	0.84	0.60	1.16	0.277	0.50	0.22	1.15	0.104
A (rs12617311)	*PLCL1*	2	895	0.99	0.80	1.23	0.954	1.05	0.82	1.33	0.714	1.03	0.75	1.43	0.838	1.12	0.68	1.84	0.649
C (rs6438424)	*IGSF11*	3	888	0.95	0.78	1.17	0.647	0.93	0.74	1.16	0.518	0.96	0.67	1.36	0.800	0.85	0.58	1.25	0.408
A (rs2013573)	*UGT2B4*	4	899	1.23	0.95	1.60	0.120	1.20	0.88	1.62	0.249	1.22	0.87	1.70	0.252	1.27	0.42	3.83	0.676
A (rs13111134)	*UGT2B4*	4	896	1.27	1.00	1.62	0.052	1.28	0.96	1.71	0.095	1.35	0.98	1.87	0.068	1.06	0.40	2.81	0.905
C (rs222003)	*GC*	4	900	1.21	0.80	1.83	0.360	1.07	0.67	1.71	0.765	1.05	0.65	1.72	0.833	2.01	0.18	22.37	0.571
C (rs222020)	*GC*	4	899	0.96	0.70	1.32	0.806	1.08	0.75	1.55	0.666	1.09	0.75	1.60	0.649	0.98	0.14	7.01	0.985
G (rs3756261)	*EGF*	4	898	1.00	0.69	1.47	0.983	1.01	0.66	1.56	0.954	1.01	0.64	1.58	0.963	1.11	0.09	14.24	0.935
T (rs757647)	*KDM3B*	5	889	0.94	0.74	1.19	0.621	0.99	0.75	1.31	0.955	1.08	0.78	1.49	0.658	0.59	0.25	1.41	0.237
G (rs7766109)	*F13A1*	6	898	1.16	0.95	1.42	0.141	1.06	0.85	1.33	0.619	1.10	0.77	1.58	0.600	1.06	0.73	1.54	0.778
A (rs4946651)	*LIN28B*	6	900	1.00	0.81	1.22	0.972	1.14	0.90	1.44	0.274	1.20	0.86	1.69	0.288	1.16	0.75	1.77	0.504
C (rs7759938)	*LIN28B*	6	899	1.04	0.83	1.30	0.718	1.18	0.92	1.52	0.180	1.31	0.95	1.81	0.104	1.05	0.59	1.88	0.861
T (rs314280)	*LIN28B*	6	888	0.99	0.80	1.22	0.918	1.14	0.89	1.44	0.301	1.18	0.84	1.67	0.334	1.17	0.74	1.83	0.505
A (rs314276)	*LIN28B*	6	874	0.97	0.78	1.21	0.791	1.10	0.86	1.41	0.445	1.15	0.83	1.59	0.398	1.08	0.63	1.85	0.789
G (rs3020394)	*ESR1*	6	899	1.24	1.00	1.54	0.049	1.17	0.92	1.49	0.202	1.07	0.78	1.47	0.687	1.74	1.05	2.88	0.033
G (rs1884051)	*ESR1*	6	900	1.22	0.99	1.52	0.068	1.14	0.89	1.45	0.291	1.04	0.76	1.43	0.812	1.67	1.00	2.80	0.051
C (rs7753051)	*IGF2R*	6	898	1.01	0.81	1.26	0.910	0.93	0.72	1.20	0.579	1.05	0.76	1.44	0.772	0.53	0.27	1.05	0.068
C (rs1079866)	*INHBA*	7	899	0.89	0.68	1.16	0.386	0.88	0.65	1.19	0.403	0.91	0.64	1.28	0.579	0.55	0.19	1.60	0.275
T (rs2288696)	*FGFR1*	8	899	1.12	0.87	1.44	0.367	1.08	0.81	1.44	0.588	1.19	0.85	1.65	0.307	0.59	0.22	1.60	0.298
A (rs10980926)	*ZNF483*	9	899	0.86	0.69	1.07	0.185	0.85	0.66	1.08	0.186	0.85	0.62	1.18	0.334	0.68	0.38	1.21	0.193
C (rs10441737)	*ZNF483*	9	880	0.81	0.65	1.02	0.068	0.79	0.61	1.01	0.058	0.79	0.57	1.09	0.144	0.60	0.33	1.07	0.083
C (rs10769908)	*STK33*	11	885	1.14	0.93	1.40	0.207	1.11	0.89	1.39	0.354	1.35	0.94	1.95	0.106	0.97	0.66	1.42	0.860
G (rs555621)	*FSHB*	11	896	1.13	0.92	1.39	0.232	1.08	0.85	1.36	0.531	1.16	0.82	1.64	0.395	1.02	0.66	1.57	0.937
A (rs11031010)	*FSHB*	11	892	1.11	0.82	1.51	0.484	1.12	0.80	1.57	0.498	1.16	0.80	1.69	0.445	0.99	0.29	3.37	0.987
C (rs1782507)	*FSHB*	11	898	1.08	0.88	1.33	0.476	1.19	0.94	1.51	0.151	1.11	0.80	1.54	0.531	1.59	1.01	2.51	0.047
A (rs6589964)	*BSX*	11	900	0.87	0.71	1.06	0.165	0.80	0.64	1.01	0.058	0.78	0.55	1.11	0.170	0.70	0.47	1.04	0.080
A (rs1544410)	*VDR*	12	896	1.18	0.96	1.45	0.121	1.20	0.95	1.53	0.125	1.28	0.92	1.78	0.150	1.26	0.79	1.99	0.332
A (rs999460)	*NKX2-1*	14	898	**1.37**	**1.11**	**1.69**	**0.003**	**1.45**	**1.14**	**1.83**	**0.002**	1.33	0.96	1.83	0.088	**2.41**	**1.53**	**3.82**	**0.0002**
A (rs4986938)	*ESR2*	14	898	0.95	0.77	1.18	0.657	0.93	0.73	1.19	0.576	0.90	0.65	1.24	0.510	0.96	0.58	1.59	0.883
A (rs2241423)	*MAP2K5*	15	895	1.21	0.93	1.57	0.160	1.13	0.83	1.52	0.434	1.13	0.80	1.59	0.480	1.30	0.50	3.38	0.597
T (rs12444979)	*GPRC5B*	16	895	**1.67**	**1.27**	**2.20**	**0.0002**	**1.56**	**1.14**	**2.13**	**0.005**	**1.59**	**1.11**	**2.27**	**0.011**	2.56	0.94	6.92	0.065
A (rs9939609)	*FTO*	16	898	1.10	0.90	1.35	0.348	1.07	0.85	1.34	0.584	1.06	0.75	1.49	0.748	1.13	0.75	1.70	0.547
A (rs12324955)	*FTO*	16	898	0.97	0.78	1.22	0.813	0.94	0.74	1.21	0.636	1.05	0.76	1.44	0.786	0.61	0.33	1.14	0.120
G (rs1398217)	*SKOR2*	18	891	0.97	0.79	1.19	0.778	1.06	0.84	1.34	0.618	0.99	0.70	1.38	0.936	1.26	0.82	1.94	0.296
G (rs2252673)	*INSR*	19	897	1.04	0.81	1.33	0.787	0.98	0.74	1.30	0.891	1.06	0.76	1.47	0.751	0.56	0.21	1.46	0.233
A (rs1073768)	*GHRH*	20	899	1.08	0.88	1.32	0.473	1.01	0.81	1.26	0.932	1.17	0.82	1.68	0.397	0.86	0.58	1.27	0.446
C (rs4633)	*COMT*	22	898	1.08	0.88	1.32	0.442	1.12	0.90	1.40	0.318	1.19	0.83	1.70	0.346	1.14	0.79	1.66	0.488
A (rs5930973)	*CD40LG*	23	893	1.10	0.73	1.65	0.658	1.10	0.69	1.74	0.690								
T (rs3092921)	*CD40LG*	23	901	1.13	0.78	1.62	0.524	1.28	0.85	1.92	0.230								

Note: Chr, chromosome; OR, odds ratio; 95%CI, 95% confidence interval of OR; L95, lower limit of 95%CI; U95, upper limit of 95%CI; *p*, significance level, significant *p*-values shown in bold.

**Table 3:** Associations of haplotypes with FGR.

Haploblocks (Genes) and Included SNPs	Haplotypes	Frequency		OR	*p*
		FGR (n = 273)	Controls (n = 631)		
H1 (*FSHB*) rs555621-rs11031010-rs1782507	ACC	0.368	0.349	1.20	0.131
	GAA	0.131	0.120	1.11	0.529
	GCA	0.308	0.290	1.02	0.881
	**ACA**	**0.194**	**0.242**	**0.71**	**0.017**
H2 (*ZNF483*) rs1098092-rs10441737	AC	0.284	0.323	0.80	0.083
	GT	0.716	0.677	1.23	0.104
H3 (*ESR1*) rs3020394-rs1884051	GG	0.333	0.289	1.15	0.268
	AA	0.667	0.711	0.87	0.235
H4 (*LIN28B*) rs4946651-rs7759938	AC	0.288	0.280	1.18	0.185
	AT	0.120	0.130	0.96	0.803
	GT	0.592	0.590	0.88	0.283
H5 (*UGT2B4*) rs2013573-rs13111134	AA	0.191	0.159	1.22	0.202
	GA	0.042	0.033	1.39	0.255
	GG	0.767	0.808	0.77	0.078
H6 (*FSHR*) rs6732220-rs4953655	GG	0.224	0.246	0.84	0.200
	GA	0.018	0.011	1.60	0.301
	CA	0.757	0.743	1.17	0.243
H7 (*LHCGR*) rs7579411-rs4953616	TC	0.276	0.286	0.93	0.580
	TT	0.156	0.169	1.10	0.534
	CT	0.568	0.546	1.02	0.896
H8 (*POMC*) rs7579411-rs4953616	AA	0.297	0.299	0.97	0.785
	AG	0.178	0.153	1.17	0.317
	GG	0.526	0.548	0.95	0.658

Note: significant values shown in bold.

**Table 4:** SNP × SNP interactions associated with FGR.

N	Models	NH	beta H	WH	NL	beta L	WL	*p*_perm_
Two-order interaction (*p* < 5 × 10^−6^)								
1	rs3020394 *ESR1* × rs7538038 *KISS1*	2	0.494	10.21	3	−0.762	20.93	<0.001
2	rs3020394 *ESR1* × rs12444979 *GPRC5B*	3	0.836	22.05	1	−0.571	13.00	<0.001
3	rs7538038 *KISS1* × rs999460 *NKX2-1*	3	0.732	23.74	1	−0.773	11.42	<0.001
Three-order interaction (*p* < 1 × 10^−8^)								
1	rs222003 *GC* × rs7538038 *KISS1* × rs999460 *NKX2-1*	5	0.871	32.84	1	−1.072	16.80	<0.001
2	rs713586 *RBJ* × rs3020394 *ESR1* × rs12444979 *GPRC5B*	5	1.194	33.03	3	−0.794	18.91	<0.001
3	rs3020394 *ESR1* × rs999460 *NKX2-1* × rs12444979 *GPRC5B*	3	0.736	14.48	3	−0.872	32.13	<0.001
4	rs11031010 *FSHB* × rs999460 *NKX2-1* × rs12444979 *GPRC5B*	4	0.801	17.78	3	−0.816	30.32	<0.001
5	rs7538038 *KISS1* × rs999460 *NKX2-1* × rs12444979 *GPRC5B*	6	0.809	29.90	3	−0.809	27.65	<0.001
Four-order interaction (*p* < 3 × 10^−12^)								
1	rs1544410 *VDR* × rs1398217 *SKOR2* × rs7579411 *LHCGR* × rs314280 *LIN28B*	8	1.547	49.59	0	-	-	<0.001
2	rs314280 *LIN28B* × rs10769908 *STK33* × rs13111134 *UGT2B4* × rs7579411 *LHCGR*	8	1.280	47.32	1	−0.665	2.77	<0.001

Note: The results were obtained using the model-based multifactor dimensionality reduction (MB-MDR) method with adjustment for covariates; NH, number of significant high risk genotypes in the interaction; beta H, regression coefficient for high-risk exposition in Step 2 analysis; WH, Wald statistic for the high-risk category; NL, number of significant low-risk genotypes in the interaction; beta L, regression coefficient for low-risk exposition in Step 2 analysis; WL, Wald statistic for the low-risk category; p_perm_, permutation *p*-value for the interaction model (1000 permutations).

**Figure 1:**
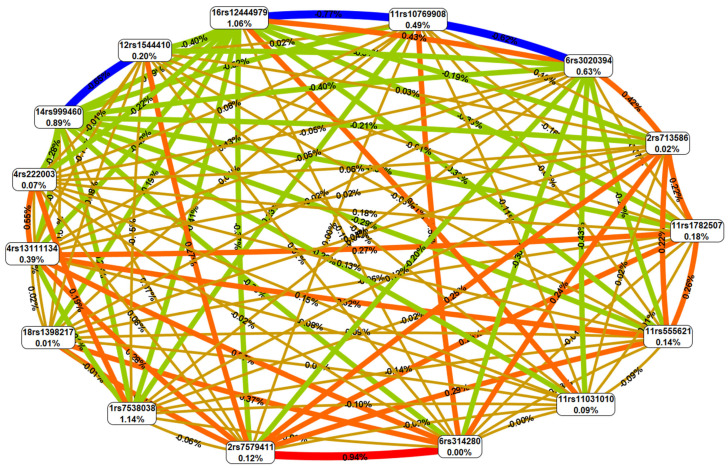
The entropy graph of SNP × SNP interactions with fetal growth restriction. The figure outlines the SNP × SNP interactions within the 2-, 3-, and 4-locus models obtained by the MB-MDR method. The polymorphisms are shown by the chromosome number and rs SNP ID. The percentage at the bottom of each SNP represents its entropy, and the percentage on each line represents the percentage of interaction between the 2 SNPs. The red and orange lines indicate a stronger and weaker synergism, respectively, brown—an independent effect of individual SNPs, green—weaker antagonism, blue—stronger antagonism.

The graph describing the interaction of 15 FGR-associated SNPs (Figure 1) demonstrates the highest indicators (% entropy) of the contribution to the FGR susceptibility of such individual loci as rs12444979 *GPRC5B* (1.14%), rs7538038 *KISS1* (1.06%), and rs999460 *NKX2-1* (0.89%) and such SNP-paired interactions as rs757647 *KDM3B-*rs314280 *LIN28B* (0.94%), rs999460 *NKX2-1*-rs1544410 *VDR* (−0.65%), rs10769908 *STK33*-rs12444979 *GPRC5B* (−0.77%), and rs3020394 *ESR1*-rs12444979 *GPRC5B* (−0.62%).

### 2.4. FGR-Significant Locus/Gene Probable Functions

In this section of the work, a detailed analysis of the functional relevance of 15 FGR-causal loci and 430 proxy SNPs was carried out, aimed at assessing the possible connection of the considered loci (n = 445) with missense mutations, epigenetic changes, gene expression and splicing regulatory effects and their involvement in FGR-related pathways.

#### 2.4.1. Missense Mutations and FGR-Linked SNPs

Three loci determining the missense mutations were discovered by us among 430 proxy SNPs such as rs4889 *KISS1* (replacement of proline with arginine in the 81 position of the KISS1 protein; “deleterious” SIFT grade), rs11676272 *ADCY3* (replacement serine with proline in the 107 position of the ADCY3 protein; “benign” SIFT grade), rs61742688 *GPRC5B* (replacement of asparagine with lysine in the 268 position of the GPRC5B protein; “benign” SIFT grade).

#### 2.4.2. Link FGR-Involved Loci with Deoxyribonucleic Acid (DNA) Epigenetic Changes

It was revealed that all 15 FGR-causal genetic variants and the absolute majority of linkage disequilibrium (LD) SNPs (381 out 430 loci, 88.60%) potentially had different regulatory potential: they were localized in DNA sites (motifs) providing communication with transcription-regulating factors (TrF) (373/445; 83.82%), regulatory-significant gene regions such as enhancers (93/445; 20.90%) and promoters (57/445; 12.81%), potentially functionally active regions of the genome (the so-called open chromatin) (105/445; 23.59%), conservative fragments of human DNA (20/445; 4.49%), and places providing DNA “dialogue” with protein regulators (31/445; 6.97%) (Table S3). The most “outstanding” regulatory potential has been demonstrated by such proxy loci as rs11865578 (LD with FGR-causal rs12444979 *GPRC5B*) [SNP is in the enhancer/promoter positions and open chromatin in 20/4 and 21 organs, respectively, including FGR-impact cultured cells (derived trophoblast (H1 BMP4 culture), endoderm(CD184+)/ectoderm(CD56+)/mesoderm(CD56+), neuronal progenitor cells(H1,H9) and neuron(H9), etc.), maternal (brain, skeletal muscle, ovary, placenta amnion, etc.) and fetal (brain, muscle, adrenal glands, etc.) organs], rs1222218 and rs58336049 (LD with rs1782507) [loci are in the promoter positions in 24 organs each, open chromatin in 49 and 47 organs, respectively, areas providing gene “dialogue” with 19 and 18 protein regulators, and 11 and 2 TrF, respectively], etc. (Table S3).

Importantly, FGR-causal SNP, rs7538038 *KISS1*, was functionally active (by modulating the activity of enhancers) in FGR-correlated organs: placenta (IDepigenome-E091), a variety of maternal organs such as brain (hippocampus middle [IDepigenome-E071], substantia nigra [IDepigenome-E074], the anterior caudate [IDepigenome-E068], the dorsolateral-prefrontal cortex [IDepigenome-E073], the germinal matrix [IDepigenome-E070]), the female skeletal muscle [IDepigenome-E108], etc., and in a multitude of fetal organs such as the brain, male (IDepigenome-E081) and female (IDepigenome-E082), muscle (IDepigenome-E089/IDepigenome-E090), kidney (IDepigenome-E086), lung (IDepigenome-E088), thymus (IDepigenome-E093), etc. Similarly, another FGR-causal locus, rs999460 *NKX2-1*, also demonstrated high functional potential (by regulating the enhancers or/and promoter activity) in disorder-linked cultured cells such as derived CD184+_endoderm (IDepigenome-E011)/CD56+_ectoderm (IDepigenome-E012)/H1_BMP4_mesendoderm (IDepigenome-E004), etc. (Table S3).

In total, 15 FGR-causal SNPs and 381 variants strongly linked to them possess regulatory influences on the 19 genes (*GPR139*, *REN*, *IQCK*, *ADCY3*, *SKOR2*, *C11orf46*, *TRIM66*, *ESR1*, *LIN28B*, *GC*, *FSHB*, *LHCGR*, *GPRC5B*, *KISS1*, *UGT2B4*, *NKX2-1*, *STK33, RBJ*, *VDR*) in the variety of FGR-related maternal/fetal organs/cell cultures (brain, muscle, ovary, placenta, amnion, trophoblast, endoderm, ectoderm, mesoderm, neuronal progenitor cells, neurons, etc.) (Table S3).

#### 2.4.3. Possible Gene Expression Regulatory Effects of FGR-Linked Polymorphisms

Three FGR-involved SNPs had possible regulatory effects on the expression (eQTL) of five genes such as *CENPO*, *ADCY3*, *RBJ* (rs713586), *C11orf46* (rs11031010), *HDHD2* (rs1398217) in peripheral blood (data of the Blood eQTL portal, Table S4). At the same time, 13 loci strongly linked with them (rs713586 and rs1398217) were also eQTL-serious for four genes in blood (*HDHD2*, *ADCY3*, *CENPO*, *RBJ*) (Table S5).

For 12 out of 15 FGR-related loci, we found sizeable effects on the level of transcriptional activity of 22 genes (*GPRC5B*, *ADCY3*, *HDHD2*, *ARL14EP*, *IER3IP1*, *CENPO*, *LIN28B*, *DNAJC27-AS1*, *KNOP1*, *REN*, *STK33*, *RBJ*, *UGT2A3P7*, *STON1-GTF2A1L*, *EFR3B*, *UGT2B4*, *FSHB*, *RP4-710M3.1*, *LINC00577*, *HDAC7*, *POMC*, *TRIM66*), including in organs involved in FGR biology such as subcutaneous (*GPRC5B*, *ADCY3*, *ARL14EP*, *CENPO*, *KNOP1*) and visceral (*KNOP1*, *DNAJC27-AS1*, *ADCY3*, *STK33*) adipose tissue, ovary (*KNOP1*, *ARL14EP*), skeletal muscle (*IER3IP1*, *HDHD2*, *STK33*, *KNOP1*), breast (*STK33, GPRC5B*), thyroid (*IER3IP1*, *TRIM66*, *STON1-GTF2A1L*, *KNOP1*, *ARL14EP*), the adrenal gland (*KNOP1, ARL14EP*), blood (*KNOP1*, *CENPO*, *DNAJC27-AS1*, *ADCY3*, *ARL14EP*), brain [hypothalamus (*GPRC5B*, *FSHB*), basal ganglia (*GPRC5B*, *ARL14EP*), cortex (*LIN28B*, *FSHB*)], etc. (Table S6). Interestingly, the risk allele for FGR-T rs12444979 *GPRC5B*,was associated (p_FDR_ ≤ 0.05) with higher *KNOP1* gene mRNA production in subcutaneous (β = 0.64) and visceral (β = 0.81) adipose tissue, skeletal muscle (β = 0.73), breast (β = 0.81), ovary (β = 1.00), thyroid (0.58), blood (β = 0.35), adrenal gland (β = 0.66), hypothalamus (β = 0.89) and basal ganglia (nucleus accumbens) (β = 0.82) of brain and lower *GPRC5B* transcription in the subcutaneous adipose (β = −0.25) (Table S6).

According to the eQTL data provided in Tables S6 and S7 of the 15 FGR-involved SNPs have 330 proxy SNPs, eQTL-substantial for 23 genes such as *EFR3B*, *ADCY3, HDHD2*, *ARL14EP*, *REN*, *CENPO*, *RP11-49K24.8*, *DNAJC27-AS1, LINC00577*, *UGT2A3P7*, *RBJ*, *FSHB*, *TRIM66*, *HACE1*, *STK33*, *IER3IP1*, *NCOA1*, *LIN28B*, *SMAD2*, *POMC*, *RP4-710M3.1*, *STON1-GTF2A1L*, *UGT2B4*. The most marked eQTL effects have been demonstrated by loci linked to FGR-associated SNPs *FSHB* (rs5556214; rs11031010; rs1782507-130 LD loci affect the expression of three genes such as *FSHB*, *RP4-710M3.1*, *ARL14EP* in the ovary), *STK33* (rs10769908) (130 LD variants are involved in transcriptional activity regulation of two genes [*STK33*, *TRIM66*] in visceral adipose and breast), *RBJ* (rs713586) (20 proxy SNPs were linked with mRNA levels of seven genes such as *NCOA1*, *EFR3B*, *CENPO*, *POMC*, *ADCY3*, *RBJ, DNAJC27-AS1* in blood, visceral/subcutaneous adipose (Table S7).

In total, 12 out of 15 FGR-impact SNPs and 330 LD variants are used essentially for mRNA formation of 27 genes: *RP11-49K24.8*, *ADCY3*, *UGT2B4, ARL14EP*, *UGT2A3P7*, *C11orf46*, *TRIM66*, *CENPO*, *STON1-GTF2A1L*, *DNAJC27-AS1*, *SMAD2*, *EFR3B*, *HACE1*, *FSHB*, *LIN28B*, *GPRC5B*, *LINC00577*, *HDAC7*, *RP4-710M3.1*, *HDHD2*, *POMC*, *NCOA1*, *KNOP1*, *RBJ*, *IER3IP1*, *REN STK33*.

#### 2.4.4. Potential Opportunity of Splicing Regulation of FGR-Correlated Loci

It was found that 7 out of 15 FGR-associated loci (46.67%) (Table S8) and 250 out of 430 SNPs in LD with them (58.14%) (Table S9) have a potential influence on the formation of alternative splicing variants of immature messenger ribonucleic acid (mRNA) (sQTL) of eight genes such as *SFTA3, ADCY3, LIN28B-AS1, ARL14EP, KNOP1, DNAJC27-AS1, STK33, KATNAL2*. Moreover, these effects were meaningful in such FGR-significant organs of the maternal organism as subcutaneous (*ADCY3, KNOP1, ARL14EP*) and visceral-omentum (*KNOP1, ARL14EP*) adipose, adrenal (*ARL14EP*) and thyroid (*KATNAL2, ARL14EP, KNOP1, SFTA3*) glands, ovary (*KNOP1*), whole blood (*ARL14EP*), skeletal muscle (*KNOP1,ARL14EP*), the brain cortex and pituitary (in both *ARL14EP*), cultured fibroblasts (*ARL14EP*), breast (mammary tissue) (*KNOP1, ARL14EP*) etc. Interestingly, FGR-risky allele T rs12444979 *GPRC5B* was correlated with low sQTL parameters of the *KNOP1* gene in subcutaneous adipose (Intron ID: 19707221:19710509:clu_17456, β = −0.52, *p* = 4.4 × 10^−10^, p_FDR_ ≤ 0.05), ovary (Intron ID: 19707221:19710509:clu_14053, β = −0.73, *p* = 2.7 × 10^−8^, p_FDR_ ≤ 0.05), and breast (mammary tissue) (Intron ID: 19707221:19710509:clu_17535, β = −0.49, *p* = 1.6 × 10^−6^, p_FDR_ ≤ 0.05) (Table S8). The 51 sQTL-impact SNPs in the above organs have been strongly linked to this locus (Table S9). Also, the largest number (116 SNPs) of sQTL-considerable loci for the *ARL14EP* gene in cultured fibroblasts, breast, visceral adipose, and adrenal gland were in disequilibrium with FGR-associated polymorphisms (as part of the haplotype and SNP interaction models) rs555621 and rs1782507 *FSHB* (Table S9). These polymorphisms themselves are also sQTL-serious for the *ARL14EP* gene in the above organs (Table S8).

#### 2.4.5. Probable FGR-Related Pathways

Based on the in silico data obtained at the previous stages of this research, it can be assumed that 39 genes are involved in predisposition to FGR due to the functional effects of 15 FGR-causal SNPs and 430 proxy loci on them (missense alterations in 3 genes [*GPRC5B, ADCY3*, *KISS1*], epigenetic transformations near 19 genes [*GC*, *IQCK*, *REN*, *ADCY3*, *C11orf46*, *ESR1*, *FSHB*, *UGT2B4*, *GPR139*, *GPRC5B*, *VDR, STK33*, *KISS1*, *LHCGR*, *LIN28B*, *NKX2-1*, *RBJ*, *SKOR2*, *TRIM66*], regulation of expression of 27 genes [*UGT2B4, ADCY3*, *UGT2A3P7*, *ARL14EP*, *TRIM66*, *C11orf46*, *STON1-GTF2A1L*, *CENPO*, *STK33*, *SMAD2*, *DNAJC27-AS1*, *POMC*, *EFR3B*, *RP4-710M3.1*, *FSHB*, *RP11-49K24.8*, *REN*, *GPRC5B*, *RBJ*, *HACE1*, *LINC00577*, *NCOA1*, *HDAC7*, *LIN28B*, *HDHD2*, *IER3IP1, KNOP1*] and coordination of splicing of 8 genes [*SFTA3, ADCY3, LIN28B-AS1, ARL14EP, KNOP1, DNAJC27-AS1, STK33, KATNAL2*]). GeneOntology enrichment analysis (released:2023-07-12) allowed us establishment of the involvement of 39 FGR-casual genes in the regulation of hormone levels (GO:0010817; FE-9.21; p_FDR_-0.029), the ovulation cycle process (GO:0042698; Fold Enrichment (FE)-47.99; p_FDR_-0.023), male gonad development (GO:0008584; FE-21.51; p_FDR_-0.020), and vitamin D metabolism (P04396; FE-89.13; p_FDR_-0.047).

**Figure 2:**
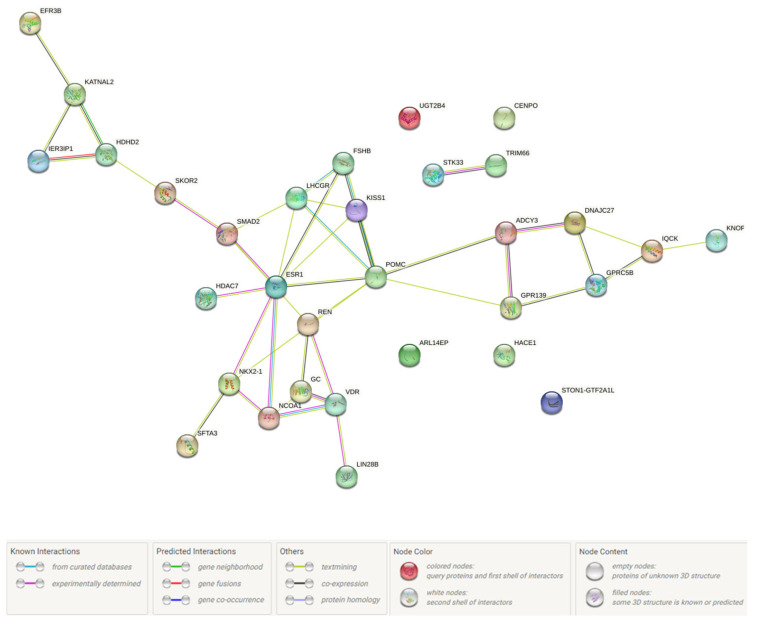
FGR-related protein–protein interaction networks inferred using the STRING resource.

**Figure 3:**
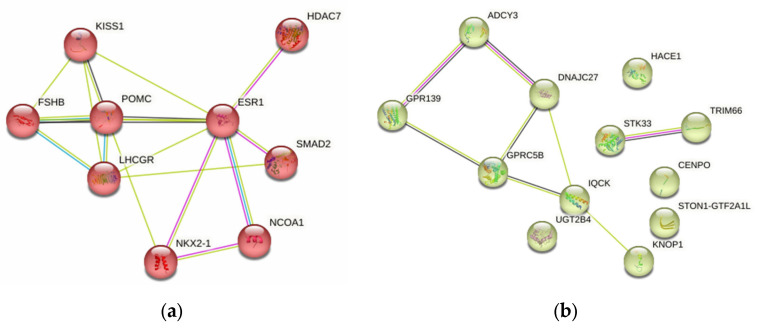

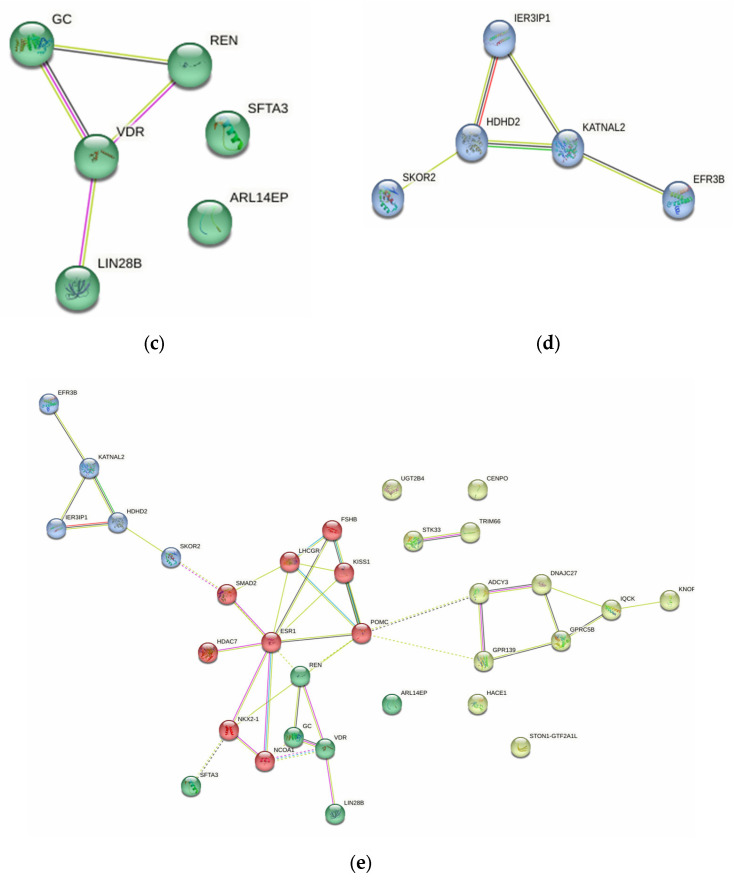
FGR-related protein–protein interaction clusters inferred using the STRING resource (four groups of PPint clusters are highlighted in color: Cluster 1, red (**a**); Cluster 2, yellow (**b**); Cluster 3, green (**c**); Cluster 4, blue (**d**); summary of four clusters (**e**)).

Using the STRING program (released: 12 July 2023), we conducted a detailed and in-depth analysis of protein–protein interplays (PPint) of 39 FGR-causal genes. The results obtained are shown in Figure 2 and Figure 3. Three substantial KEGG pathways for this 39-gene set such as ovarian steroidogenesis (hsa04913; p_FDR_-0.024), the estrogen signaling pathway (hsa04915; p_FDR_-0.024), the cAMP signaling pathway (hsa04024; p_FDR_-0.042) were detected by the STRING database. PPints were subdivided (clustered) into four groups (Figure 3e). The first cluster, shown in Figure 3a, combining nine proteins (HDAC7, ESR1, FSHB, KISS1, LHCGR, NCOA1, NKX2-1, POMC, SMAD2) has been involved in multiple hormone-mediated signaling and other molecular/reactome pathways such as the ovulation cycle (GO:0042698; p_FDR_-8.71 × 10^−7^), male gonad development (GO:0008584; p_FDR_-8.54 × 10^−6^), ovarian follicle development (GO:0001541; p_FDR_-0.0017), regulation of hormone levels (GO:0010817; p_FDR_-0.0016), positive regulation of transcription by RNA polymerase II (GO:0006357; p_FDR_-0.0035), regulation of cell communication (GO:0010646; p_FDR_-0.0050), peptide hormone biosynthesis (HSA-209952; p_FDR_-0.0106), FOXO-mediated transcription of oxidative stress (HSA-9615017; p_FDR_-0.0144), ADORA2B mediated anti-inflammatory cytokine production (HSA-9660821; p_FDR_-0.0106), DNA-binding transcription factor binding (GO:0140297; p_FDR_-0.0475), the phospholipase C-activating G protein-coupled receptor signaling pathway (GO:0007200; p_FDR_-0.0052), positive regulation of the nitrogen compound metabolic process (GO:0051173; p_FDR_-0.0215), etc. The second cluster, presented in Figure 3b, consists of twelve proteins (TRIM66, GPRC5B, KNOP1, ADCY3, STON1-GTF2A1L, CENPO, DNAJC27, GPR139, UGT2B4, HACE1, IQCK, STK33) linked with BMI-impact parameters (according to Human Phenotype (Monarch) data) such as waist circumference (EFO:0004342; p_FDR_-0.0034) and BMI (EFO:0004340; p_FDR_-0.0012). The third cluster, demonstrated in Figure 3c, incorporates six proteins (GC, VDR, ARL14EP, LIN28B, REN, SFTA3) engaged in vitamin D (calciferol) metabolism (HSA-196791; p_FDR_-0.0132). The fourth cluster, displayed in Figure 3d, unites five interaction proteins (KATNAL2, EFR3B, HDHD2, IER3IP1, SKOR2) associated with the Yip1 domain and the Yos1-like pathway (STRING local network data; CL:34722; p_FDR_-0.0185).

**Figure 4:**
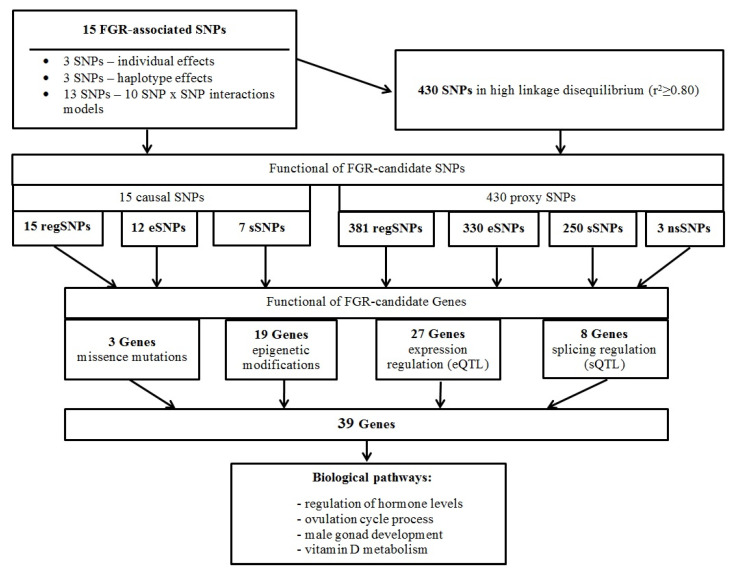
Study result outline.

The result outline of this study is presented in Figure 4.

### 2.5. Syntropic Effects of mAAM-Involved Genes in FGR, mAAM, Pre-Pregnancy mBMI and Offspring BW

We conducted a comparative analysis of the data obtained in this paper with the results of our previous studies devoted to the study of associations of the same list of mAAM-related loci in the same population (women of the Russian ethnic group from Central Russia) with maternal AAM and BMI (mBMI) [36], BW [40], and uterus benign proliferative diseases (uterine leiomyoma (UL) [33]; endometrial hyperplasia (EH) [31]; endometriosis [32]) in order to identify common genetic factors underlying these reproductively significant phenotypes. As a result of this comparative analysis (Table S10), FGR-related polymorphisms associated with mAAM, mBMI and BW in this population were established (Table S10, [31,32,33,36,40]): out of 15 FGR-associated loci, 11 variants were associated with mBMI/BW (73.33%), including 5 SNPs with BW (33.33%), 2 SNPs-mBMI (13.33%) and 4 SNPs with both BW and mBMI (26.67%); only 2 FGR-associated loci were mAAM-associated (13.33%).

Common genetic determinants for the three phenotypes—FGR, BW and mBMI—were four loci including rs314280 *LIN28B*, rs3020394 *ESR1*, rs555621 *FSHB* and rs999460 *NKX2-1* (Table S10). Along with this, ten out of fifteen FGR-associated mAAM-significant polymorphisms (66.67%) also determined susceptibility to uterus benign proliferative diseases: seven SNPs were UL-linked (46.67%) and six SNPs were EH- and endometriosis- associated (40.00% each). Interestingly, three loci including rs4374421 *LHCGR*, rs12324955 *FTO*, rs1782507 *FSHB* (13.63%) have been associated simultaneously with FGR and two different proliferative uterine diseases mentioned above, and three *FSHB* gene polymorphisms (rs11031010; rs555621; rs1782507) were correlated with FGR and all three uterine diseases under consideration (Table S10). These data convincingly demonstrate significant similarity in genetic determinants (mAAM-related loci) FGR and BW/mBMI (73.33%) on the one hand, and FGR and uterus benign proliferative diseases (66.67%) on the other hand.

**Figure 5:**
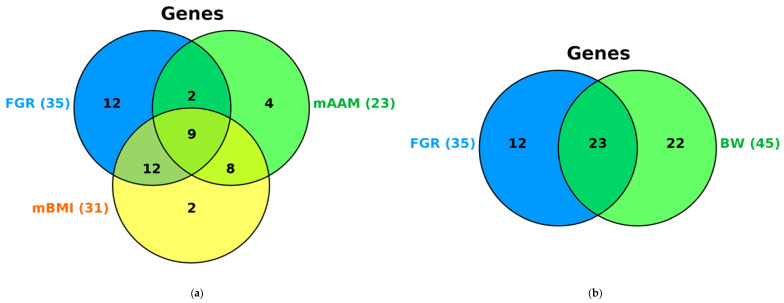
Venn diagram characterizing the syntropic effects of mAAM-involved genes in FGR and maternal AAM (mAAM) and pre-pregnancy BMI (mBMI) (**a**), FGR and offspring BW (**b**) (due to the functionality of FGR-,BW-,AAM- and BMI-associated mAAM-involved loci (15 SNPs [the data of this work], 13 SNPs (mAAM) and 14 SNPs (mBMI) [36] and 22 SNPs (offspring BW) [40], respectively) and proxy variants).

Amid FGR-correlated genes, a large majority—60.00%—were mBMI-linked and only 31.43% were mAAM-associated; meanwhile, interestingly, 25.71% of genes (*LIN28B*; *HACE1*; *ADCY3*; *LIN28B-AS*; *EFR3B3*; *RBJ*; *LHCGR*; *STON1-GTF2A1L*; *POMC*) were enabled in the genetic determination of all three examined phenotypic sings (FGR, mAAM, mBMI) [Figure 5a, genes functionally affected by mAAM-involved loci such as 15 SNPs (FGR; the data of this research), 22 SNPs (BW [40]), 13 SNPs (AAM [36]), 14 SNPs (BMI [36]) and proxy loci]. Meanwhile, 67.74% of mBMI-linked genes and 47.83% of mAAM-associated genes were FGR-correlated genes (Figure 5a). Among the FGR-associated genes, 65.71% were BW-influential, and amid the BW-linked genes, 51.11% were FGR-affecting (Figure 5b). The common FGR- and BW-relating genes were *LIN28B*; *SFTA3*; *GC*; *ADCY3*; *LIN28B-AS1*; *LHCGR*; *ARL14EP*; *IQCK*; *GPRC5B*; *CENPO*; *ESR1*; *DNAJC27-AS1*; *EFR3B*; *FSHB*; *KNOP1*; *GPR139*; *HACE1*; *RBJ*; *KISS1*; *NKX2-1*; *REN*; *POMC*; *UGT2B4.*

## 3. Discussion

This is the first study to find the association of mAAM-involved gene polymorphisms and FGR susceptibility risk. Three SNPs independently (rs999460 *NKX2-1*; rs7538038 *KISS1*; rs12444979 *GPRC5B*), one haplotype *FSHB* gene (rs555621*rs11031010*rs1782507) and ten SNPs-interworking models of 13 loci were FGR-implicated. FGR-correlated mAAM-related 15 polymorphisms with 430 proxy variants were functionally meaningful to 39 genes participating in the regulation of hormone levels, the ovulation cycle process, male gonad development and vitamin D metabolism.

The present study revealed associations of rs12444979 *GPRC5B* with FGR both independently (the T allele of this SNPs was risky for FGR, OR = 1.56–1.67) and as part of SNP interactions models (50% of FGR risk models included this SNPs). Also, in our previously performed genetic study in this population (Russian women of Central Russia), the relationship of maternal rs12444979 *GPRC5B* (as part of interloci “dialogue” models) with offspring birth weight (BW) [40] was shown. rs12444979 *GPRC5B* was AAM-bounded [41,42] and BMI-involved [43,44,45,46], wherein the T allele of this SNP correlated with a later AAM [41], and an alternative genetic variant for it, the C allele, was associated with increased BMI [43,44,46]. Hence, the maternal AAM-boosting/BMI-lowering allele rs12444979 *GPRC5B* was an FGR risk factor. It is believed that low pre-pregnancy weight and low weight gain during pregnancy in a woman are linked to an increased FGR risk [11,15,17].

Our in silico materials pointed to significant SNP-eQTL and SNP-sQTL correlations: the FGR risk allele T rs12444979 *GPRC5B* was associated with higher *KNOP1* gene mRNA production in multiple FGR-impact organs such as adipose (subcutaneous; visceral), skeletal muscle, ovary, thyroid, blood, adrenal gland, brain (hypothalamus, basal ganglia), lower *GPRC5B* transcription in subcutaneous adipose and low *KNOP1* gene splicing level (together with 51 proxy SNPs) in adipose (subcutaneous), ovary, breast. The *GPRC5B* gene (it is placed on 12p13.1) encodes a receptor protein coupled with G proteins [47], playing an essential role in many different FGR-significant factors (insulin resistance, inflammation, cell growth/differentiation/apoptosis, etc.) due to intracellular signaling via pathways such as mitogen-activated protein kinase (MAPK)-c-Jun NH2-terminal kinase (JNK), transforming growth factor *beta* (TGF-*β*), interferon *gamma* (IFNγ), cyclic adenosine monophosphate (cAMP), nuclear factor *κ*B (NF-*κ*B), signal transducer and activator of transcription (STAT3), focal adhesion kinase (FAK)/Src family kinases (SRC), and signaling cascades [48,49,50]. There are experimental data (obtained using transfected cell line models) on the key role of *GPRC5B* in metabolic stress processes (due to the modulation of interaction with phosphorylated sphingomyelin synthase 2) underlying lipid-generated insulin resistance [49]. The fundamental value of GPRC5B in the development of a chronic inflammatory process and the formation of insulin resistance in adipose as a result has been proven on a *GPRC5B*-deficient mice model [51], which indicated the critical role of this gene in the regulation of insulin-susceptible organ (muscles, adipose, central nervous system) metabolism. These processes are essential in the pathophysiology of FGR [7]. The *KNOP1* gene (for which SNP rs12444979 *GPRC5B* was sQTL-significant) encoded a nuclear protein such as lysine-rich nucleolar protein 1 interacting with a zinc finger 106 protein [51]. This protein has been involved in the regulation of particular developmental genes (e.g., *TSG118*, *TSPYL*) by chromatin rebuilding [52].

As a result of this study, correlation between rs7538038 *KISS1* and FGR was shown (allele G, OR = 0.56–0.61). This SNP was functionally active (due to the *KISS1* gene enhancers activity modulating) in FGR-correlated organs such as placenta, maternal brain (hippocampus, substantia nigra, cortex, etc.) and skeletal muscle, a multitude of fetal organs (brain male/female, muscle, kidney, lung, etc.) (our in silico materials). In previous studies, rs7538038 *KISS1* was linked with AAM (the G allele was a genetic factor of early AAM) [53], central precocious puberty in girls (allele G was risky for central precocious puberty) [54], BW and endometrial hyperplasia (as part of inter-genic interworkings, [31,40], respectively). So, we can generalize that the AAM-reducing allele G rs7538038 *KISS1*, also associated with central precocious puberty of girls, is an FGR-protective factor.

The *KISS1* gene encodes the kisspeptin protein (KP), which is cleaved into shorter, biologically active molecules—kisspeptins (KP-54, KP-14, KP-13; KP-10), which have a biological effect by activating the G-protein-coupled receptor 54 (GPR54), also known as the KISS receptor-1 [55]. The KP/GPR54 system plays an important physiological role in neuroendocrine regulation of reproduction by influencing the hypothalamic–pituitary–gonadal axis. In addition, it affects fertility, implantation processes, and stages of the menstrual cycle [56,57]. In placental tissues, *KISS1* mRNAs and KPs are found in the syncytiotrophoblast and, to a lesser extent, in the cytotrophoblast, whereas KISS1R is expressed in the syncytiotrophoblast and villous and invasive extracellular trophoblast [58]. There are data on the implication of KPs in placentation regulation. In particular, KP-10 produced by trophoblast cells in the first trimester inhibits cell migration [58]. It is assumed that a decrease in the expression of *KISS1* and *KISS1R* may be correlated with a violation of placentation and FGR development: a number of studies have recorded a decrease in the level of KPs in pregnant women with FGR compared with women with physiological pregnancy [59,60,61]. In addition, pregnant women with FGR are characterized by a weaker increase in the level of KPs throughout pregnancy and a lower level of KPs at the end of the first and third trimester of gestation [59].

The FGR-causal locus of rs999460 *NKX2-1* increases this pregnancy complication risk (allele A, OR = 1.37–2.41) and determines susceptibility to FGR in various intergenic interactions. This SNP has a high functional potential: it was involved in the enhancer or/and promoter activity regulating disorder-linked cultured cells such as the derived CD184+_endoderm, the CD56+_ectoderm, the H1_BMP4_mesendoderm, etc., determining the nucleic acids binding with such 5TrFs as STAT, AIRE, Foxa, Arid5a, Pax-45 and controlling *SFTA3* gene splicing in the thyroid (FGR-risk allele A was linked with high *SFTA3* sQTL). Previously, associations of rs999460 *NKX2-1* with AAM [53], female BMI [36], endometrial hyperplasia [31], and offspring BW [40] were presented. Interestingly, the AAM-boosting A allele rs999460 *NKX2-1* [53] determined a high risk of FGR (OR = 1.37–2.41, our data) and was associated with a low BW [40].

*NKX2-1* encodes a protein—factor transcription regulation (called TTF1 [thyroid transcription factor 1]) that communicates and “enables” (activates) promoters of the several thyroid-related hormone/protein genes such as thyroperoxidase, thyroglobulin, and the thyroid-stimulating hormone receptor [https://www.genecards.org/ (accessed on 21 September 2023)]. Besides this, the above hormones/proteins suppress TrF NR1D1 production and inhibit, due to this, the activity of genes important for processes of gluconeo- and adipo-genesis, bile acid/lipid metabolism, and inflammatory cell responses [62]. According to literature materials, transcription factor TTF1 (protein product of the NKX2-1 gene) is intensively synthesized in the process intrauterine development in several embryonic organs such as brain (hypothalamus, diencephalon, ventral forebrain, etc.), lungs, thyroid, etc. [63]. Thyroid-implicated TTF1 production is vital for both the early stages of thyroid formation and embryonic development/growth overall [63]. TTF1 with other several thyroid-associated TrFs (FOXE1/HHEX/PAX8) is collectively expressed in the process of thyroid formation in progenitor/mature follicular cells of this gland, providing thyroid formation/growth/differentiation/function/homeostasis at a necessary level [63]. In TTF1 absence cases, thyroid progenitor cells may undergo apoptosis leading to their disappearance in the early stages of embryonic growth, resulting in a significant decrease in the number/mass of thyroid follicular cells; its formation is disrupted, and degradation occurs [64]. The structural–functional disturbances of the thyroid may underlie various pregnancy complications such as premature childbirth, birth of preterm newborns and infants with low BW [63], which correlate with FGR outright [7]. Epidemiological data indicate the presence of hypothyroxinemia at the 30th week of the gestation period in more than half of all infants with low BW [64].

Importantly, there is a unified result obtained for all three genes (*KISS1*, *NKX2-1*, *GPRC5B*) strongly associated with FGR: allelic variants of these genes are associated with an increased risk of FGR (the A allele, rs999460 *NKX2-1* [OR = 1.37–2.41], and the T allele, rs12444979 *GPRC5B* [1.56–1.67]), which in previous studies showed a link with late menarche [41,53] and low BW (rs999460 *NKX2-1* [40]) and adult BMI (rs12444979 *GPRC5B* [43,44]); and vice versa, the G allelic variant of the *KISS1* gene (rs7538038) is correlated with a low risk of FGR (OR = 0.56–0.63), according to earlier studies, associated with the early menarche [53]. It should be noted that the association of low maternal BMI with an increased risk of FGR is now believed to be proven and is not in doubt [2,11,15]. There is also no doubt about the connection between genetic determinants of late/early AAM and low/high BMI, which has been repeatedly proven in previous research [34,35,37,38,39]. In accordance with the above scientific facts, the relationship we establish between the genetic factors of late/early mAAM and low/high FGR risk corresponds to the generally accepted ideas in this field at the present time: later menarche─>low BMI─>high FGR risk.

In this work, we convincingly demonstrate a significant similarity in genetic determinants (mAAM-related loci) FGR and BW/mBMI (73.33%) on the one hand and FGR and uterus benign proliferative diseases (66.67%) on the other hand. In our earlier study, the proportion of “common” polymorphisms for BW and mBMI (36.36%) and proliferative uterine diseases (UL (40.90%), EH (45.45%), endometriosis (36.36%) [40]) in general corresponded to the data of this study. Nevertheless, if the common genetic determinants for BW/mAAM/mBMI were four loci (rs1073768 *GHRH*; rs4374421 *LHCGR*; rs4633 *COMT*; rs4946651 *LIN28B* [62]), then in this study, no such “common” genetic factors were identified for FGR/BW/mAAM/mBMI, but “common” genetic variants for FGR/BW/mBMI were completely different from the above list of SNPs (4 SNPs-rs314280 *LIN28B*; rs3020394 *ESR1*; rs555621 *FSHB*; rs999460 *NKX2-1*). There are also significant differences in the list of “common” genetic determinants for FGR and three benign uterine diseases (3 SNPs *FSHB*-rs11031010; rs555621; rs1782507; materials of this study) and for BW and the same three uterine diseases (3 SNPs-rs12324955 *FTO*; rs4374421 *LHCGR*; rs1782507 *FSHB*; materials of the previous study [40]). These data show that despite the presence of “common” heredity (due to mAAM-related factors) between FGR and BW/mBMI/UL/EH/endometriosis and BW and mBMI/UL/EH/endometriosis, the specific genetic determinants (polymorphisms) underlying this differ significantly, despite the significant similarity of FGR and BW in the mAAM-linked polymorphisms that define them (60.00%).

The significant similarity in the genetic “architecture” of FGR and BW shown in our study (a high percentage, more than 50%, of common genes such as *LIN28B*; *SFTA3*; *GC*; *ADCY3*; *LIN28B-AS1*; *LHCGR*; *ARL14EP*; *IQCK*; *GPRC5B*; *CENPO*; *ESR1*; *DNAJC27-AS1*; *EFR3B*; *FSHB*; *KNOP1*; *GPR139*; *HACE1*; *RBJ*; *KISS1*; *NKX2-1*; *REN*; *POMC*; *UGT2B4*) is consistent with the biomedical logic (as a rule, a fetus/newborn with a low body weight is the “basis” of the FGR group) and the literature data on this topic [7,29]. For example, the *LIN28B* gene (according to our data, this gene is essential for both FGR and BW) controls the formation of powerful specific regulators of the cell cycle (the let-7 family of miRNA) [65] and involved in the pubertal growth/development timing in girls/boys [66]. There is persuasive experimental evidence that LIN28B-let-7 regulating the insulin/phosphoinositide 3-kinases (PI3Ks)/mammalian target of the rapamycin (mTOR) pathway (by modulating the insulin receptor (INSR), insulin-like growth factor1 receptor (IGF1R), insulin receptor substrate 2 (IRS2) effects) is a midland controller of glucose metabolism (changing insulin resistance and glucose tolerance) in mammals [67]. Several let-7 targets such as Hmga2, Myc, Igf2bp1, Kras are well-known controllers of glucose/insulin metabolism and mammalian size of body [68]. The polymorphisms of this gene, according to the results of multiple investigations, have been associated with adult height/weight/BMI [69,70,71,72]. Thus, our materials and literature data indicate that LIN28B may be one of the potentially relevant causal genes for both BW and FGR.

## 4. Materials and Methods

### 4.1. Study Design/Subjects

**Figure 6:**
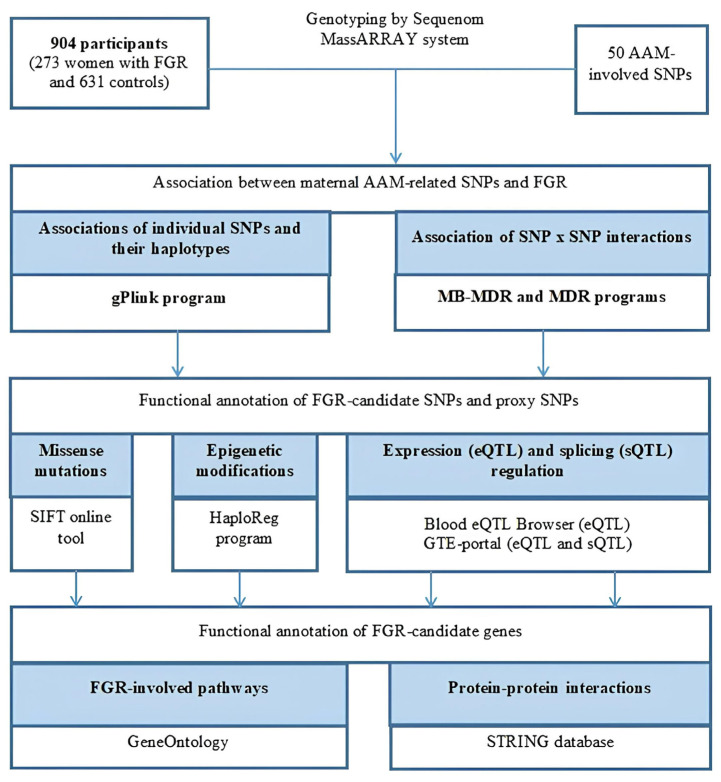
Study design.

The outline of study design is shown in Figure 6. This case (FGR)–control (FGR-free) study included 904 women (273 FGR and 631 control) in the third trimester of gestation examined/treated in the Departments of Obstetrics (Belgorod Regional Clinical Hospital, Russia) during 2008–2017. All participants provided informed consent (form signed in person) prior to the start of this study. The medical ethics commission of both the Belgorod State University and the Belgorod Regional Clinical Hospital approved protocol/design of this study.

When forming the sample (FGR/FGR free), we used (a) a 24–41-week single pregnancy ending in a live birth, (b) Russian nationality (self-reported), (c) birthplace inside of Russia (Central region) [73,74] as inclusion parameters (criteria). Such parameters as age of <16 years, multifetal pregnancy, fetal/newborn congenital defects, maternal uterine congenital disturbance, and delivery at <24 weeks were used as exclusion criteria.

Clinical information, newborn growth/weight values, fetal ultrasound study (fetometry) results (analysis carried out using device TOSHIBA XARIO SSA-660A) were used for FGR diagnosis [75,76]. The degrees of FGR were determined by comparing nomograms with photometry data [77]. In the studied FGR group (n = 273), the first degree was diagnosed in 48.72% (n = 133) of participants, the second degree in 42.49% (n = 116), the third degree in 8.79% (n = 24). The pregnancy control group (n = 631) did not have FGR.

The participants of present research have previously been involved in other genetic studies of disorders/outcomes of pregnancy (newborn weight, preeclampsia, FGR) (detailed data on the results of these studies are contained in previous publications [40,75,76,78,79,80,81,82]). Table 1 presents the biological/medical characteristics of the FGR/FGR free cohorts formed.

### 4.2. Genetic Laboratory Analysis

To identify associations of mAAM genes with FGR, we specifically selected SNPs in these genes considering the ensuing four criteria [31,32,33,36,40]: (a) a previously acknowledged link with mAAM (generalizing literature data are given in Table S11, [34,35,36,41,42,44,45,53,66,70,83,84,85,86,87,88,89,90,91,92,93,94,95,96,97,98,99,100,101,102,103,104,105,106,107,108,109,110,111,112,113,114,115,116,117,118,119,120,121,122,123,124,125,126,127]); (b) a previously demonstrated correlation with mAAM-related traits such as anthropometric phenotypes (height; weight; BMI; obesity), vit.D metabolic traits, etc. (Table S11); (c) functionality validity such as epigenetic alterations, connection with transcription of genes, etc. (this assessment was carried out in silico using Haploreg data [128], the obtained estimates of SNPs functionality are presented in Table S12); (d) effective (minor) allele frequency of ≥5% (among Europeans). As a result, the application of the above criteria made it possible to include 50 functionally significant SNPs that we previously used in the study of genetic factors of AAM [36], newborn weight [40], and female reproductive system pathology [31,32,33]. In total, 40 loci among 50 selected for this research were mAAM-appertain (13 SNPs in genome-wide association studies (GWAS) and 27 SNPs in associative studies) and 10 SNPs (rs222003/rs222020 *GC*; rs1884051/rs3020394 *ESR1*; rs4633 *COMT*; rs12324955 *FTO*; rs1544410 *VDR*; rs3756261 *EGF*; rs7766109 *F13A1*; rs2252673 *INSR*); although they were not mAAM-significant, they were involved in mAAM-important pathways such as hormone/vitamin D traits (disorder), etc. (Table S11), and had potential functional capabilities (Table S12). Additionally, 15 out 50 SNPs have been linked with several anthropometric phenotypes (Table S11).

To collect 4–5 mL of peripheral (venous) blood, vacuum tubes (containing ethylene diamine tetra acetic acid (EDTA)) were used, from which genomic DNA was subsequently extracted (the “classical” method of isolation based on stepwise phenol–chloroform–ethanol procedures was used [129]). The resulting DNA was stored in kelvinator (temperature −80 °C). For multiplex genotyping on the Sequenom device (experimental genotyping procedures were carried out at the “Medical Genomics” Core Facility of Tomsk National Research Medical Center of the Russian Academy of Sciences (Tomsk, Russia), “working” samples with a DNA level (concentration) of 5–10 ng in one microliter were prepared (the Nanodrop-2000 measuring device (spectrophotometer) was used). To estimate the experimental data obtained, such indicators of SNP genotyping quality control were used as the call rate of at least 90% and the duplicate (blank) check success rate of at least 99% (90%) [33,130]. One SNP, rs11724758 *FABP2,* did not meet the above quality requirements (call rate = 86.13%) and was excluded from further statistical genetic analysis. Overall, 49 SNPs corresponded to all of the abovementioned quality requirements.

### 4.3. Statistical Genetic Analysis

Analysis of the HWE for every polymorphism in FGR/FGR free cohorts was performed [131,132]. Association between FGR risk and mAAM-connected SNPs was detected by logistic regression with the help of such software tools as gPlink (version 1.07) [133] (for individual loci [four genetic models such as allelic, recessive, additive, dominant [134] were tested] and SNP haplotypes), MB-MDR (version 2.6) [135,136] and multifactor dimensionality reduction (MDR) [137,138] (for SNP interworking) taking into account multi-test calibration (permutation was performed) [139,140] and covariates (such as age, BMI before the current pregnancy, number of gravidity and induced abortions in the anamnesis, the presence in anamnesis of arterial hypertension, FGR and preeclampsia according to the information granted in Table 1). The following parameters, p_perm_, were declared as statistically meaningful: for individual SNPs, p_perm_ ≤ 0.0125 (multi-test calibration based on Bonferroni correction [0.05/4 according to the quantity of examined genetic models] was performed); for SNPs haplotypes, p_perm_ ≤ 0.050; for models of SNPs interactions, p_perm_ < 0.001. It seems important that when choosing FGR-related SNP interworking models for permutation testing in order to obtain more reliable results, we used additional Bonferroni corrections (the potential quantity of 49-locus feasible recombination taken into account). As a result, significance level parameters *p* for models of different multi-locus levels were derived (used as “threshold indicators” for choosing FGR-related SNP interworking models for permutation testing) such as 2 SNP interworking -<0.05/1176 = 4 × 10^−5^; 3 SNP interworking -<0.05/18,424 = 3 × 10^−6^; 4 SNP interworking -<0.05/211,876 = 2 × 10^−7^ [40]. The subjects amounted to n = 904 (case = 273/control = 631), with a scheduled study power of ≥80% allowing identification of differences at the level of OR_additive_1.33–1.56, OR_dominant_1.59–1.63, OR_recessive_1.61–4.71. Power indicators for FGR-linked loci were computed by the Quanto tool [141].

*FGR-significant locus/gene probable functions.* We explored FGR-correlated loci and LD SNPs (r^2^ was not less than 0.80 [142,143]) from the standpoint of their possible functionality [144,145,146]. For the purpose of in-depth/comprehensive analysis of materials on this issue, based on the positively proven in extensive (including GWAS) genetic research with an in silico approach [147,148,149,150], six different contemporary bioinformatic programs/resources were utilized, such as (a) GTE Consortium data [151], (b) HaploReg [128], (c) GeneOntology knowledge base [152], (d) STRING [153], (e) Blood eQTL resource [154], and (f) SIFT [155].

## 5. Conclusions

The present study proves the link between mAAM-involved gene polymorphisms with FGR mediated by functional effects of FGR-associated SNPs. The data obtained expand the understanding of the medico-biological significance of maternal age at menarche genes in the formation of pregnancy complications.

## Data Availability

The data generated in the present study are available from the corresponding author upon reasonable request.

## Supplementary Materials

The following supporting information can be downloaded at: https://www.mdpi.com/article/10.3390/ijms25052647/s1.
