# Supplementary material for: Maternal Age at Menarche Genes Determines Fetal Growth Restriction Risk
Source: Int J Mol Sci. 2024 Feb 24;25(5):2647. doi: 10.3390/ijms25052647 (PMC10932237; doi:10.3390/ijms25052647)
Supplement: Supplementary file 1 [file ijms-25-02647-s001.zip › +Suppl Table S1.pdf]

Supplementary Table S1. Distribution of alleles and genotypes frequencies of 49 SNPs in the study group.

Minore allele (SNP)	Gene	Chr	Genotypes ($A_1A_1/A_1A_2/A_2A_2$)	Minor allele frequency	H_o	H_e	P
			FGR (n=273)				
T (rs1514175)	*TNNI3K*	1	48/121/102	0.400	0.447	0.480	0.256
T (rs466639)	*RXRG*	1	4/54/214	0.114	0.199	0.202	0.762
G (rs7538038)	*KISS1*	1	9/75/188	0.171	0.276	0.284	0.668
C (rs713586)	*RBJ*	2	59/121/91	0.441	0.447	0.493	0.139
A (rs2164808)	*POMC*	2	59/141/73	0.474	0.517	0.499	0.628
A (rs7589318)	*POMC*	2	20/122/131	0.297	0.447	0.417	0.310
C (rs4374421)	*LHCGR*	2	18/127/125	0.302	0.470	0.422	0.062
T (rs7579411)	*LHCGR*	2	49/136/86	0.432	0.502	0.491	0.804
C (rs4953616)	*LHCGR*	2	19/115/138	0.281	0.423	0.404	0.549
G (rs6732220)	*FSHR*	2	12/108/152	0.243	0.397	0.368	0.247
G (rs4953655)	*FSHR*	2	11/100/162	0.223	0.366	0.347	0.484
A (rs12617311)	*PLCL1*	2	30/115/124	0.325	0.428	0.439	0.678
C (rs6438424)	*IGSF11*	3	61/133/78	0.469	0.489	0.498	0.808
A (rs2013573)	*UGT2B4*	4	7/90/176	0.191	0.330	0.308	0.327
A (rs13111134)	*UGT2B4*	4	8/111/153	0.234	0.408	0.358	0.027
C (rs222003)	*GC*	4	1/35/237	0.068	0.128	0.126	1.000
C (rs222020)	*GC*	4	2/58/212	0.114	0.213	0.202	0.549
G (rs3756261)	*EGF*	4	1/39/232	0.075	0.143	0.139	1.000
T (rs757647)	*KDM3B*	5	7/115/147	0.240	0.428	0.365	0.004
G (rs7766109)	*F13A1*	6	71/135/67	0.507	0.495	0.500	0.904
A (rs4946651)	*LIN28B*	6	45/133/94	0.410	0.489	0.484	0.901
C (rs7759938)	*LIN28B*	6	22/112/137	0.288	0.413	0.410	1.000
T (rs314280)	*LIN28B*	6	40/133/95	0.397	0.496	0.479	0.611
A (rs314276)	*LIN28B*	6	25/112/131	0.302	0.418	0.422	0.885
G (3020394)	*ESR1*	6	38/109/126	0.339	0.399	0.448	0.079
G (1884051)	*ESR1*	6	35/112/126	0.333	0.410	0.444	0.220
C (rs7753051)	*IGF2R*	6	20/127/125	0.307	0.467	0.426	0.120
C (rs1079866)	*INHBA*	7	5/81/187	0.167	0.297	0.278	0.381
T (rs2288696)	*FGFR1*	8	7/98/168	0.205	0.359	0.326	0.136
C (rs10980926)	*ZNF483*	9	25/106/142	0.286	0.388	0.408	0.458
C (rs10441737)	*ZNF483*	9	24/105/141	0.283	0.389	0.406	0.458
C (rs10769908)	*STK33*	11	57/148/63	0.489	0.552	0.500	0.112
G (rs555621)	*FSHB*	11	48/142/81	0.439	0.524	0.493	0.325
A (rs11031010)	*FSHB*	11	4/63/204	0.131	0.233	0.228	1.000
C (rs1782507)	*FSHB*	11	39/122/112	0.366	0.447	0.464	0.517
A (rs6589964)	*BSX*	11	51/142/80	0.447	0.520	0.494	0.462
A (rs1544410)	*VDR*	12	40/138/94	0.401	0.507	0.480	0.380
A (rs999460)	*NKX2-1*	14	45/121/106	0.388	0.445	0.475	0.308
A (rs4986938)	*ESR2*	14	33/121/118	0.344	0.445	0.451	0.790
A (rs2241423)	*MAP2K5*	15	8/86/177	0.188	0.317	0.306	0.691
T (rs12444979)	*GPRC5B*	16	11/78/183	0.184	0.287	0.300	0.425
A (rs9939609)	*FTO*	16	54/133/86	0.441	0.487	0.493	0.902
A (rs12324955)	*FTO*	16	20/113/139	0.281	0.415	0.404	0.764
G (rs1398217)	*SKOR2*	18	40/136/95	0.399	0.502	0.479	0.526
G (rs2252673)	*INSR*	19	6/101/164	0.209	0.373	0.330	0.042
A (rs1073768)	*GHRH*	20	61/140/70	0.483	0.517	0.499	0.627
C (rs4633)	*COMT*	22	64/133/75	0.480	0.489	0.499	0.717
A (rs5930973)	*CD40LG*	23	4/28/236	0.067	0.105	0.125	0.022
T (rs3092921)	*CD40LG*	23	2/43/228	0.086	0.158	0.157	1.000
			Control group (n=631)				
T (rs1514175)	*TNNI3K*	1	100/277/248	0.382	0.443	0.472	0.128
T (rs466639)	*RXRG*	1	16/124/487	0.124	0.198	0.218	0.027
G (rs7538038)	*KISS1*	1	31/247/348	0.247	0.395	0.372	0.134
C (rs713586)	*RBJ*	2	135/293/200	0.448	0.467	0.495	0.170

A (rs2164808)	*POMC*	2	129/307/189	0.452	0.491	0.495	0.872
A (rs7589318)	*POMC*	2	68/241/318	0.301	0.384	0.421	0.036
C (rs4374421)	*LHCGR*	2	65/254/285	0.318	0.421	0.434	0.454
T (rs7579411)	*LHCGR*	2	118/329/173	0.456	0.531	0.496	0.090
C (rs4953616)	*LHCGR*	2	48/264/308	0.290	0.426	0.412	0.437
G (rs6732220)	*FSHR*	2	42/237/347	0.256	0.379	0.381	0.835
G (rs4953655)	*FSHR*	2	39/233/353	0.249	0.373	0.374	0.915
A (rs12617311)	*PLCL1*	2	72/265/289	0.327	0.423	0.440	0.364
C (rs6438424)	*IGSF11*	3	149/294/173	0.481	0.477	0.499	0.294
A (rs2013573)	*UGT2B4*	4	10/181/435	0.161	0.289	0.270	0.076
A (rs13111134)	*UGT2B4*	4	15/211/398	0.193	0.338	0.312	0.039
C (rs222003)	*GC*	4	2/67/558	0.057	0.107	0.107	1.000
C (rs222020)	*GC*	4	5/138/484	0.118	0.220	0.208	0.182
G (rs3756261)	*EGF*	4	2/90/534	0.075	0.144	0.139	0.566
T (rs757647)	*KDM3B*	5	33/245/342	0.251	0.395	0.376	0.239
G (rs7766109)	*F13A1*	6	140/307/178	0.470	0.491	0.498	0.748
A (rs4946651)	*LIN28B*	6	106/304/218	0.411	0.484	0.484	1.000
C (rs7759938)	*LIN28B*	6	54/243/331	0.280	0.387	0.403	0.323
T (rs314280)	*LIN28B*	6	95/306/219	0.400	0.494	0.480	0.504
A (314276)	*LIN28B*	6	61/252/293	0.309	0.416	0.427	0.568
G (rs3020394)	*ESR1*	6	49/268/309	0.292	0.428	0.414	0.440
G (rs1884051)	*ESR1*	6	49/266/312	0.290	0.424	0.412	0.498
C (rs7753051)	*IGF2R*	6	54/273/299	0.304	0.436	0.423	0.509
C (rs1079866)	*INHBA*	7	22/186/418	0.184	0.297	0.300	0.790
T (rs2288696)	*FGFR1*	8	24/186/416	0.187	0.297	0.304	0.598
C (rs10980926)	*ZNF483*	9	64/269/293	0.317	0.430	0.433	0.854
C (rs10441737)	*ZNF483*	9	66/267/277	0.327	0.438	0.440	0.927
C (rs10769908)	*STK33*	11	137/289/191	0.456	0.468	0.496	0.168
G (rs555621)	*FSHB*	11	101/309/215	0.409	0.494	0.483	0.620
A (rs11031010)	*FSHB*	11	11/126/484	0.119	0.203	0.210	0.442
C (rs1782507)	*FSHB*	11	69/298/258	0.349	0.477	0.454	0.252
A (rs6589964)	*BSX*	11	149/307/171	0.483	0.490	0.499	0.632
A (rs1544410)	*VDR*	12	79/294/251	0.362	0.471	0.462	0.665
A (rs999460)	*NKX2-1*	14	56/284/286	0.316	0.454	0.433	0.267
A (rs4986938)	*ESR2*	14	73/298/255	0.355	0.476	0.458	0.338
A (rs2241423)	*MAP2K5*	15	16/169/439	0.161	0.271	0.270	1.000
T (rs12444979)	*GPRC5B*	16	9/130/484	0.119	0.209	0.209	0.850
A (rs9939609)	*FTO*	16	111/300/214	0.418	0.480	0.486	0.743
A (rs12324955)	*FTO*	16	58/243/325	0.287	0.388	0.409	0.204
G (rs1398217)	*SKOR2*	18	94/315/211	0.406	0.508	0.482	0.211
G (rs2252673)	*INSR*	19	27/200/399	0.203	0.320	0.323	0.805
A (rs1073768)	*GHRH*	20	140/304/184	0.465	0.484	0.498	0.521
C (rs4633)	*COMT*	22	140/296/190	0.460	0.473	0.497	0.228
A (rs5930973)	*CD40LG*	23	1/75/549	0.062	0.120	0.116	0.502
T (rs3092921)	*CD40LG*	23	1/95/532	0.077	0.151	0.143	0.163

Note: Chr – chromosome; H_o – observed heterozygosity; H_e – expected heterozygosity; A1 – minor allele; A2 – major allele; P – level of significance.
