# Supplementary material for: Maternal Age at Menarche Genes Determines Fetal Growth Restriction Risk
Source: Int J Mol Sci. 2024 Feb 24;25(5):2647. doi: 10.3390/ijms25052647 (PMC10932237; doi:10.3390/ijms25052647)
Supplement: Supplementary file 1 [file ijms-25-02647-s001.zip › +Suppl Table S10 ref.pdf]

Supplementary Table S10. Association of FGR-associated mAMM-involved SNPs with several maternal/offspring phenotypes in the studied population.

Chr (1)	SNP (2)	Gene (3)	FGR (4)	Maternal/offspring phenotypes					
				biological			disorder		
				age at menarche* (5)	BMI* (6)	offspring BW (7)**	uterine leiomyoma (8)***	endometrial hyperplasia (9)****	endometriosis (10)*****
1	rs1514175	*TNNI3K*				**yes**	**yes**		**yes**
1	rs466639	*RXRG*							
1	rs7538038	*KISS1*	**yes**			**yes**		**yes**	
2	rs713586	*RBJ*	**yes**			**yes**	**yes**		**yes**
2	rs2164808	*POMC*		**yes**	**yes**				
2	rs7589318	*POMC*				**yes**	**yes**		
2	rs4374421	*LHCGR*		**yes**	**yes**	**yes**	**yes**	**yes**	**yes**
2	rs7579411	*LHCGR*	**yes**	**yes**			**yes**		**yes**
2	rs4953616	*LHCGR*			**yes**		**yes**		
2	rs6732220	*FSHR*						**yes**	
2	rs4953655	*FSHR*			**yes**		**yes**	**yes**	
2	rs12617311	*PLCL1*		**yes**	**yes**				
3	rs6438424	*3q13.32*		**yes**				**yes**	**yes**
4	rs2013573	*UGT2B4*				**yes**	**yes**	**yes**	
4	rs13111134	*UGT2B4*	**yes**					**yes**	
4	rs222003	*GC*	**yes**			**yes**			
4	rs222020	*GC*					**yes**		
4	rs3756261	*EGF*							
5	rs757647	*KDM3B*				**yes**			
6	rs7766109	*F13A1*						**yes**	
6	rs4946651	*LIN28B*		**yes**	**yes**	**yes**	**yes**		
6	rs7759938	*LIN28B*		**yes**				**yes**	**yes**
6	rs314280	*LIN28B*	**yes**		**yes**	**yes**			

6	rs314276	*LIN28B*		**yes**			**yes**	**yes**	
6	rs3020394	*ESR1*	**yes**		**yes**	**yes**			
6	rs1884051	*ESR1*							
6	rs7753051	*IGF2R*					**yes**		
7	rs1079866	*INHBA*				**yes**			
8	rs2288696	*FGFR1*							
9	rs10980926	*ZNF483*					**yes**	**yes**	**yes**
9	rs10441737	*ZNF483*				**yes**		**yes**	**yes**
11	rs10769908	*STK33*	**yes**	**yes**			**yes**		
11	rs555621	*FSHB*	**yes**		**yes**	**yes**	**yes**	**yes**	**yes**
11	rs11031010	*FSHB*	**yes**				**yes**	**yes**	**yes**
11	rs1782507	*FSHB*	**yes**			**yes**	**yes**	**yes**	**yes**
11	rs6589964	*BSX*		**yes**		**yes**		**yes**	**yes**
12	rs1544410	*VDR*	**yes**		**yes**		**yes**		**yes**
14	rs999460	*NKX2-1*	**yes**		**yes**	**yes**		**yes**	
14	rs4986938	*ESR2*				**yes**		**yes**	
15	rs2241423	*MAP2K5*					**yes**		
16	rs12444979	*GPRC5B*	**yes**			**yes**			
16	rs9939609	*FTO*							
16	rs12324955	*FTO*				**yes**	**yes**	**yes**	**yes**
18	rs1398217	*SKOR2*	**yes**		**yes**				
19	rs2252673	*INSR*		**yes**					
20	rs1073768	*GHRH*		**yes**	**yes**	**yes**			**yes**
22	rs4633	*COMT*		**yes**	**yes**	**yes**	**yes**	**yes**	
23	rs5930973	*CD40LG*							
23	rs3092921	*CD40LG*							

Note: **yes**: associated.

* Ponomarenko et al., 2019 [36];** Reshetnikova et al., 2023 [40];*** Ponomarenko et al., 2021 [33];**** Ponomarenko et al., 2020 [31];***** Ponomarenko et al., 2020 [32]
