# Supplementary material for: Maternal Age at Menarche Genes Determines Fetal Growth Restriction Risk
Source: Int J Mol Sci. 2024 Feb 24;25(5):2647. doi: 10.3390/ijms25052647 (PMC10932237; doi:10.3390/ijms25052647)
Supplement: Supplementary file 1 [file ijms-25-02647-s001.zip › +Suppl Table S11.pdf]

Supplementary Table S11. The literature data about associations of the studied polymorphisms with age at menarche and some menarche-related traits.

Chr	SNP	Gene/Region	Phenotype	Association (significance) (associated allele/genotype)	Reference
1	rs1514175	*TNNI3K*	Age at menarche<br>**Body mass index**<br>**Body mass index**<br>Body mass index	−12.2 d ($p=4.0 \times 10^{-7}$) (A)<br>**0.07 kg/m² ($p=8 x10^{-14}$) (A)**<br>**0.06 unit ($p=3 x10^{-11}$) (A)**<br>0.20 kg/m² ($p=7.7\times 10^{-4}$) (A)	[41]<br>**[44]**<br>**[83]**<br>[84]
1	rs466639	*RXRG*	**Age at menarche**<br>Body mass index<br>Age at menarche<br>**Age at menarche**	**-4.2 w ($p= 1 x10^{-13}$) (T)**<br>increasing (p= 0.011) (T)<br>-4.1 w (p= 0.023) (T)<br>**0.08 unit ($p= 2 x10^{-24}$) (C)**	**[34]**<br>[34]<br>[85]<br>**[35]**
1	rs7538038	*KISS1*	Age at menarche	-0.061 y (p_{adj}=0.012) (G)	[53]
2	rs713586	*RBJ*	Age at menarche<br>**Body mass index**	11.7 d ($p=8.4 \times 10^{-7}$) (T)<br>**0.14 kg/m² ($p= 6 x10^{-22}$) (C)**	[41]<br>**[44]**
2	rs2164808	*POMC*	Age at menarche	0.046 y (p_{adj}=0.038) (T)	[53]
2	rs7589318	*POMC*	Age at menarche	0.048 y (p_{adj}=0.047)(A)	[53]
2	rs4374421	*LHCGR*	Age at menarche	0.060 y (p_{adj}=0.017) (C)	[53]
2	rs7579411	*LHCGR*	Age at menarche	0.058 y (p_{adj}=0.013)(T)	[53]
2	rs4953616	*LHCGR*	Age at menarche	-0.065 y (p_{adj}=0.006) (T)	[53]
2	rs6732220	*FSHR*	Age at menarche<br>Age at menarche	0.17 y (p=0.0018,p_{bonf}>0.05)(G) *<br>0.34 y ($p= 3 x10^{-5}$) epistasis	[86]
2	rs4953655	*FSHR*	Age at menarche<br>Age at menarche	0.17 y (p=0.0023,p_{bonf}>0.05)(G) *<br>0.34 y ($p= 3 x10^{-5}$) epistasis	[86]
2	rs12617311	*PLCL1*	**Age at menarche**<br>Age at menarche	**-3.0 w ($p= 6 x10^{-13}$) (A)**<br>-4.7 w (p=0.004) (A)	**[34]**<br>[85]
3	rs6438424	*IGSF11*	**Age at menarche**<br>Height adult<br>Age at menarche<br>Age at menarche	**-2.7 w ($p= 1 x10^{-13}$) (A)**<br>-0.013 Zscore (p=0.0004) (A)<br>-3.7 w (p=0.008) (A))<br>0.17 y (p_{perm}=0.005) (A))	**[34]**<br>[34]<br>[85]<br>[36]
4	rs2013573	*UGT2B4*	Age at menarche	0.060 y (p_{adj}=0.019) (C)	[53]
4	rs13111134	*UGT2B4*	Age at menarche<br>Age at menarche	0.055 y (p_{adj}=0.042) (G)<br>0.431y (p_{bonf}=0.044) (A)	[53]<br>[42]
4	rs222003	*GC*	Plasma levels of 25(OH)D and	(p>0.05)	[87]

			$1,25(OH)_2D$<br>Compression strength index (male)	(p>0.05)	[88]
4	rs222020	*GC*	Plasma 25(OH)D concentrations<br>Compression strength index (male)<br>Adolescent idiopathic scoliosis	(p=0.004)<br>(p=0.0042)<br>(p<0.05)	[89]<br>[88]<br>[90]
4	rs3756261	*EGF*	Birth weight	lower (p<0.0001) (A)<br>(4 snps haplotype)	[91]
5	rs757647	*KDM3B*	**Age at menarche**<br>Age at menarche	**-2.4w(p=5.4x10^{-8},p$_{bonf}$>0.05)(A)***<br>-4.2 w (p=0.003) (A)	**[34]**<br>[85]
6	rs7766109	*F13A1*	Body mass index,<br>pressure insulin response,<br>triglycerides,<br>high density lipoprotein cholesterol in PCOS	(p=0.013)<br>(p=0.015)<br>(p=0.001)<br>(p=0.012)	[92]
6	rs4946651	*LIN28B*	**Age at menarche**	**0.085 y (p= 3 x10^{-8}) (A)**	**[93]**
6	rs7759938	*LIN28B*	**Age at menarche**<br>**Age at menarche**<br>Height adult<br>Age at menarche<br>**Height**<br>**Late pubertal growth**<br>**Age at menarche**<br>Tanner stage<br>Tanner stage (boys)<br>Tanner stage (girls)<br>Height adult<br>Height adult<br>Pubertal growth<br>Age at menarche in PCOS	**0.09 y (p= 7 x10^{-9}) (C)**<br>**6.4 w (p= 5 x10^{-60}) (C)**<br>-0.042 Zscore (p=8.7 x10^{-18}) (T)<br>8.3 w (p= 4 x10^{-6}) (C)<br>**-0.045 unit (p= 8 x10^{-31}) (T)**<br>**0.11 unit (p= 4 x10^{-9}) (C)**<br>**0.12 unit (p= 8 x10^{-110}) (C)**<br>-0.080 (p=3.42 x10^{-8}) (C)<br>-0.063 (p=0.012) (C)<br>-0.088 (p=5.57 x10^{-7}) (C)<br>increasing (p=8.69 x10^{-18}) (C)<br>0.042 cm (p= 2 x10^{-6}) (C)<br>(p=5x10^{-11})<br>earlier (p=0.006) (T)	**[94]**<br>**[34]**<br>[34]<br>[85]<br>**[95]**<br>**[96]**<br>**[35]**<br>[35]<br>[35]<br>[35]<br>[35]<br>[97]<br>[98]<br>[99]
6	rs314280	*LIN28B*	**Age at menarche**<br>**Age at menarche**<br>Age at menarche (EA)	**0.086 y (p= 2 x10^{-8}) (T)**<br>**1.2 m (p=2 x10^{-14}) (T)**<br>0.08 y (p=2 x10^{-8}) (T)	**[93]**<br>**[100]**

			Age at menarche (Asians)	0.13 y (p= 0.0012) (T)	[101]<br>[101]
6	rs314276	*LIN28B*	**Age at menarche**<br>Height<br>Breast development in girls<br>Voice breaking<br>Height growth in girls<br>**Puberty onset**	**- 0.14 y (p= 4 x10^{-16}) (C)**<br>-0.37 cm (p=3.6 x10^{-7}) (C)<br>earlier (p = 0.001)<br>earlier (p = 0.006)<br>faster tempo (p= 0.00008)<br>**0.08 unit (p= 2 x10^{-8}) (C)**	**[66]**<br>[66]<br><br><br><br>**[70]**
6	rs3020394	*ESR1*	Age at menarche<br>Hip fractures	-0.12 y (p=0.0081,p_{bonf}>0.05)(A)*<br>OR=1.66 (p=0.0004)	[86]<br>[102]
6	rs1884051	*ESR1*	Age at menarche<br>Hip fractures<br>Body weight,<br>body mass index ,<br>waist-hip ratio,<br>fat body mass,<br>body fat percentage<br>obesity in men<br>Type 2 diabetes	-0.13 y (p=0.0035,p_{bonf}>0.05)(A)*<br>OR=1.46 (p=0.0004)<br>lower (p = 0.007) (A),<br>(p = 0.003) (A) ,<br>(p= 0.011) (A) ,<br>(p= 0.010) (A),<br>(p= 0.040) (A),<br>decreased (p=0.04) (A)<br>(p=0.011)	[86]<br>[102]<br>[103]<br><br><br><br><br><br>[104]
6	rs7753051	*IGF2R*	Age at menarche<br>Age at menarche<br>Age at menarche	0.14 y (p=0.001, p_{bonf}>0.05) (T) *<br>0.34 y (p= 3 x10^{-5}) epistasis<br>0.500 y (p=0.046,p_{bonf}>0.05)(A)*	[86]<br>[86]<br>[42]
7	rs1079866	*INHBA*	**Age at menarche**<br>Height adult<br>**Age at menarche**<br>Tanner stage<br>Tanner stage (girls)<br>Height adult<br>Age at menarche	**3.9 w (p=6 x10^{-14}) (G)**<br>-0.016 Zscore (p=0.015) (C)<br>**0.07 unit (p=9 x10^{-24}) (G)**<br>-0.054 (p=0.005) (G)<br>-0.058 (p=0.015) (G)<br>increasing (p=0.015) (G)<br>0.13 y (p=0.037) (G)	**[34]**<br>[34]<br>**[35]**<br>[35]<br>[35]<br>[35]<br>[105]
8	rs2288696	*FGFR1*	Age at menarche	-0.058 y (p_{adj}=0.033) (G)	[53]
9	rs10980926	*ZNF483*	**Age at menarche**	**2.5 w (p= 4 x10^{-11}) (A)**	**[34]**

			Height adult<br>Age at menarche	-0.015 Zscore (p=0.0017) (G)<br>2.6 w (p= 0.047) (A)	[34]<br>[85]
9	rs10441737	*ZNF483*	**Age at menarche (EA)**	**-2.78 unit (p= 4 x10^{-15}) (T)**	**[106]**
11	rs10769908	*STK33*	Age at menarche	9.6 d (p=7.8× 10^{-5}) (T)	[41]
11	rs555621	*FSHB*	Age at menarche	0.059 y (p_{adj}=0.0011) (C)	[53]
11	rs11031010	*FSHB*	Age at menarche<br>PCOS	0.079 y (p_{adj}=0.0037) (A)<br>(p=4.27 × 10^{-8})	[53]<br>[107]
11	rs1782507	*FSHB*	Age at menarche	0.046 y (p_{adj}=0.021) (T)	[53]
11	rs6589964	*BSX*	**Age at menarche**<br>Height adult	**-2.7 w (p= 2 x10^{-12}) (A)**<br>-0.014 Zscore (p=0.0025) (A)	**[34]**<br>[34]
12	rs1544410	*VDR*	Vitamin D deficiency in children<br>Vitamin D vs testosterone level, free androgen index in women<br>23% of the variation in serum 25-hydroxyvitamin D concentrations in children<br>Testosterone level and free androgen index in women<br>Osteoporosis<br>Bone mineral density at the lumbar spine in girls with adolescent idiopathic scoliosis<br>Adolescent idiopathic scoliosis	OR=2.33 (p=0.041) (G)<br>(p<0.05)<br>(p<0.05)<br>p<0.002<br>OR=0.61-0.70 (p<0.05)<br>lower levels (p<0.05) (TT)<br>(p<0.05)	[108]<br>[109]<br>[110]<br>[109]<br>[111]<br>[112]<br>[90]
14	rs999460	*NKX2-1*	Age at menarche	0.046 y (p_{adj}=0.039) (A)	[53]
14	rs4986938	*ESR2*	Age at menarche	-7.0 m (p=0.005) (G)	[113]
15	rs2241423	*MAP2K5*	Age at menarche<br>**Body mass index**<br>Obesity<br>Body mass index<br>Children obesity	13.1 d (p=6.1× 10^{-6}) (A)<br>**0.13 kg/m^2 (p= 1 x10^{-18}) (G)**<br>OR = 0.79 (p= 0.029) (A)<br>−0.092 unit (p = 0.028) (A)<br>(p<0.005)	[41]<br>**[44]**<br>[114]<br>[114]

			Obesity<br>Adulthood body mass index<br>Body mass index	OR=1.34 (0.001) (G)<br>(p<0.05)<br>0.11 kg/m^2 (p=0.028) (G)	[115]<br>[90]<br>[116]<br>[84]
16	rs12444979	*GPRC5B*	Age at menarche<br>**Body mass index**<br>Body mass index<br>Body mass index (EA)<br>Body fat mass (EA)	13.5 d (p=2.6× 10^{-4}) (T)<br>**0.17 kg/m^2 (p=3 x10^{-21}) (C)**<br>(p<0.05)<br>(p=0.014)<br>(p=0.002)	[41]<br>**[44]**<br>[46]<br>[45]<br>[45]
16	rs9939609	*FTO*	**Age at menarche**<br>Body mass index<br>**Body mass index**<br>**Body mass index**<br>**Obesity**<br>Obesity<br>Obesity<br>Obesity<br>Obesity<br>Body mass index<br>Extreme obesity	**-2.1 w (p= 3 x10^{-8}) (A)**<br>increasing (p= 6.3 x10^{-17}) (A)<br>**0.33 kg/m^2 (p= 4 x10^{-51}) (A)**<br>**0.36 kg/m^2 (p= 3 x10^{-35}) (A)**<br>**OR=1.25 (p= 1 x10^{-20}) (A)**<br>OR=9.86 (p=0.026) (AA)<br>OR=2.03 (p=0.002) (AA)<br>OR=1.72 (p=0.009) (AA)<br>OR = 1.39 (p < 0.01) (A)<br>increasing (p=0.0059) (A)<br>OR=1.46 (p=1.42 x10^{-11}) (A)	**[34]**<br>[34]<br>[117]<br>[118]<br>[119]<br>[120]<br>[121]<br>[122]<br>[123]<br>[124]<br>[125]
16	rs12324955	*FTO*	Age at menarche	-0.15 y (p=0.0057,p_{bonf}>0.05)(A)*	[86]
18	rs1398217	*SKOR2*	**Age at menarche**	**-2.7 w (p= 2 x10^{-13}) (G)**	**[34]**
19	rs2252673	*INSR*	PCOS<br>PCOS	OR=1.32 (p=0.006) (G)<br>(p<0.05)	[126]<br>[127]
20	rs1073768	*GHRH*	Age at menarche	-0.046 y (p_{adj}=0.045) (C)	[53]
22	rs4633	*COMT*	Age at menarche	0.13 y (p=0.0097,p_{bonf}>0.05) (T) *	[86]
X	rs5930973	*CD40LG*	Age at menarche	0.101 y (p_{adj}=0.0067) (A)	[53]

X	rs3092921	*CD40LG*	Age at menarche	0.074 y (p_{adj}=0.037) (C)	[53]

Note: The data from the GWAS are highlighted in bold.
* putative candidate loci age at menarche (crude p<0.05, corrected for multiple comparisons p_{bonf}>0.05).
